# Rhesus macaques with an *OPA1* mutation demonstrate features of autosomal dominant optic atrophy

**DOI:** 10.1073/pnas.2509165123

**Published:** 2026-04-15

**Authors:** Tracy N. Jaggers, Ana Ripolles-Garcia, Ala Moshiri, Brett D. Story, Jun Wang, Rui Chen, Lucy G. Moore, Leandro B. C. Teixeira, Jaeho Shim, Ana C. Raposo, Maria Isabel Casanova, Sophie M. Le, Sangwan Park, Laura J. Young, Soohyun Kim, Karolina P. Roszak, Vanessa Ureno, Paige M. Karpinen, Nayeli Echeverria, Monica Ardon, Brian C. Leonard, Marguerite Knipe, Eliza Bliss-Moreau, Brad Fortune, J. Timothy Stout, Jeffrey Rogers, Nicholas Marsh-Armstrong, Sara M. Thomasy

**Affiliations:** ^a^Department of Surgical and Radiological Sciences, School of Veterinary Medicine, University of California, Davis, Davis, CA 95616; ^b^Department of Ophthalmology and Vision Science, School of Medicine, University of California, Davis, Sacramento, CA 95817; ^c^Department of Molecular and Human Genetics, Baylor College of Medicine, Houston, TX 77030; ^d^Human Genome Sequencing Center, Baylor College of Medicine, Houston, TX 77030; ^e^Comparative Ocular Pathology Laboratory of Wisconsin, School of Veterinary Medicine, University of Wisconsin-Madison, Madison, WI 53706; ^f^Department of Psychology, California National Primate Research Center, University of California, Davis, Davis, CA 95616; ^g^Legacy Devers Eye Institute, Portland, OR 97210; ^h^Cullen Eye Institute, Baylor College of Medicine, Houston, TX 77030

**Keywords:** optic neuropathy, nonhuman primate, retinal ganglion cell, autosomal dominant optic atrophy, OPA1

## Abstract

Autosomal dominant optic atrophy (ADOA) is an inherited optic neuropathy primarily caused by mutations in *OPA1*. We identified and defined a spontaneous nonhuman primate (NHP) model of ADOA using rhesus macaques heterozygous for a missense mutation (*OPA1*A8S). With ocular examinations, ophthalmic imaging, electroretinography, histopathology, immunohistochemistry, and transmission electron microscopy (TEM), we documented retinal nerve fiber layer (RNFL) thinning, retinal ganglion cell (RGC) loss and dysfunction, OPA1 mislocalization, and reduced axonal mitochondrial density in affected macaques. Our investigation revealed substantial phenotypic variability among affected macaques, shedding light on the pathogenesis of ADOA. The retinas were evaluated using techniques such as spectral-domain optical coherence tomography and fundus photography facilitating observation of structural changes in the retina and optic nerve. Thinning of the RNFL and optic nerve head degeneration, hallmark features of ADOA, were observed in affected macaques. Decreased RGC function in the *OPA1* heterozygotes was demonstrated with pattern electroretinography. Histopathological analysis and immunohistochemical staining of postmortem retinal tissue suggested RGC loss in the papillomacular bundle, with reduced OPA1 and mitochondria in the RGC axons, indicating dysfunctional mitochondrial dynamics and reduced function consistent with ADOA. Ultrastructural changes were evident with TEM including dysmorphic mitochondria, axonal loss, myelin disruption, and hypertrophic astrocytic processes. The observed similar pattern of RGC loss and dysfunction coupled with phenotypic heterogeneity in our NHP model reflects the clinical variability observed in human ADOA patients indicating that therapeutic interventions in this foveate model will likely translate to the human condition.

## Significance

Optic neuropathies due to heritable diseases are a common cause of blindness in humans, but limited therapies currently exist for the vision loss that occurs from them. One such condition is autosomal dominant optic atrophy (ADOA), an inherited optic neuropathy primarily caused by mutations in *OPA1*. We identified and defined a spontaneous NHP model of ADOA using rhesus macaques heterozygous for a missense mutation (*OPA1*A8S). With ocular examinations, imaging, electrophysiology, and microscopic examination of retinal tissues, we identified RGC loss and dysfunction, optic nerve atrophy, as well as mislocalization of OPA1 in the retina. This NHP model of ADOA closely recapitulates the human disease and will be an ideal model to test novel therapeutic interventions.

Autosomal dominant optic atrophy (ADOA) is an inherited optic neuropathy that affects approximately 3 in 100,000 people worldwide (1–3). This condition is caused by genetic mutations that result in degeneration of retinal ganglion cells (RGCs), resulting in progressive, bilateral vision impairment that can lead to blindness (1). Mutations in the *optic atrophy 1* (*OPA1*) gene, which encodes a mitochondrial dynamin-like GTPase, are associated with 65 to 90% of ADOA cases (4, 5). The OPA1 protein functions as a regulator of mitochondrial dynamics, including maintaining oxidative phosphorylation and cellular energy production as well as mitochondrial processes such as fusion and maintenance of the mitochondria structure (6, 7). Therefore, *OPA1* mutations can affect complex mitochondrial networks (8) which results in mitochondrial instability, generation of reactive oxygen species (ROS), and ultimately apoptosis. Unmyelinated axons of RGCs have an abundance of mitochondria that are vulnerable to ATP impairment by *OPA1* mutations (9). Patients typically present with temporal pallor of the optic nerve head (ONH) and decreased retinal nerve fiber layer (RNFL) thickness particularly in the papillomacular bundle (10, 11). These changes lead to a slowly progressive vision impairment, with oral idebenone being the only treatment reported to provide some visual improvement (8, 12).

Currently, most research on glaucomatous and inherited optic neuropathy is conducted using rodent models. While mice offer the advantage of relatively easy genetic manipulation and short breeding time, many rodent models do not fully recapitulate the physiology or phenotypes observed in human patients. Specifically, mice with *OPA1* mutations exhibit a relatively mild phenotype in comparison to that observed in many human patients (6, 13). Furthermore, anatomical, and physiological differences between the mouse and human retina, lamina cribrosa, and optic nerve limit the relevance of the mouse when studying optic neuropathies. Most murine RGCs highly differ from midget and parasol cells, which comprise the great majority of RGCs in humans and nonhuman primates (NHPs) and contribute to parvocellular and magnocellular visual streams (14). Such differences in the relative balance of RGC subtypes between primates and rodents are likely to be highly consequential in terms of investigating RGC pathology given their subtype-specific susceptibility to damage and regenerative capacity (15–18).

Given the prominent papillomacular pathology observed in humans with ADOA (19), a foveate species similar to humans would provide an optimal model for studying its etiopathogenesis and treatment. Here, we describe an NHP model with RGC dysfunction and loss secondary to a heterozygous missense mutation that results in an alanine to serine alteration in amino acid 8 of exon 1 (A8S) of the OPA1 protein. We report the genetic screening, identification of the causal mutation, pedigree of inheritance, ocular examination, imaging, electroretinographic, and histological findings of this *OPA1*A8S mutation in rhesus macaques. In aggregate, these NHPs heterozygous for the *OPA1*A8S mutation demonstrate a variable phenotype similar to humans with ADOA.

## Results

### The *OPA1* Mutation Is Prevalent in the California National Primate Research Center (CNPRC) Colony.

**Fig. 1.**
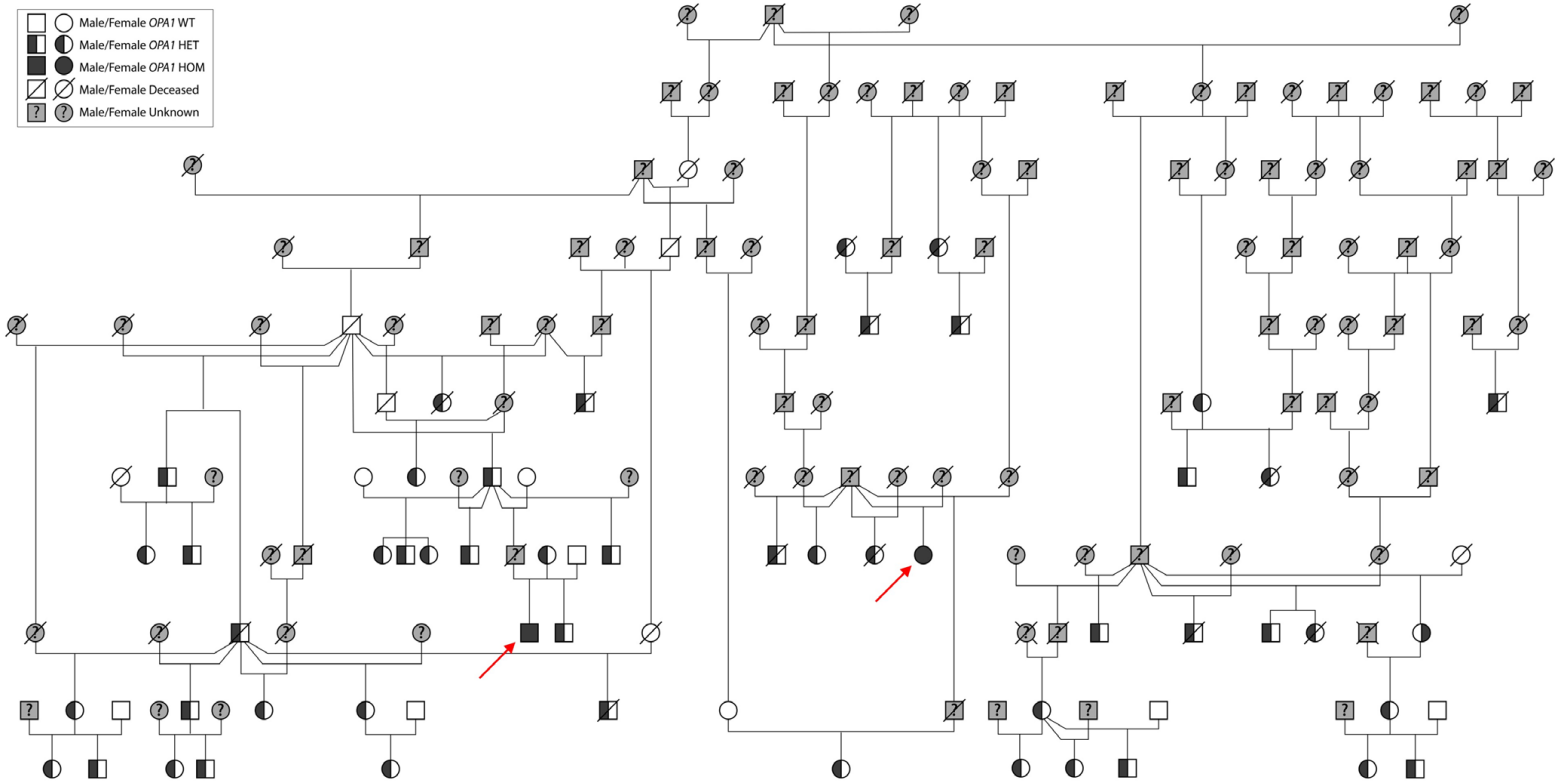
Pedigree of the *OPA1*A8S mutation in rhesus macaques demonstrates a dominant inheritance pattern. Fifty *OPA1* heterozygotes (black–white), eight WTs (white), and two mutant homozygotes (black) were identified (red arrows); NHPs in gray have an unknown genotype. Like humans with ADOA, this pedigree displays an autosomal dominant pattern of inheritance for the *OPA1*A8S mutation. Macaques no longer living within the colony are identified with a crossed line. Males are represented by squares, and females by circles.

By liftOvering the rhesus variants to the orthologous human position and screening the variants in the Human Gene Mutation Database (HGMD), we identified rhesus macaques carrying a missense mutation of *OPA1*, NM_015560.2:c.22G > T(p.ala8ser). This missense mutation was reported in a human patient with ADOA and has been observed in another patient submitted from GeneDx (20). This mutation changes alanine to serine in the 1st exon within the mitochondrial targeting region of *OPA1*. Consistently, this variant is absent in the Genome Aggregation Database (gnomAD), indicating it is very rare in the human population. This substitution has intermediate deleteriousness prediction by *in silico* prediction software, such as “Damaging” by Sorting Intolerant From Tolerant (SIFT), “Benign” by Polymorphism Phenotyping v2 HumVar (Polyphen2 HVAR), a rare exome variant ensemble learner (REVEL) score of 0.62, and a Combined Annotation Dependent Depletion (CADD v1.6) PHRED score of 23. The variant changes a nucleotide that is conserved in vertebrates with a phyloP100way_vertebrate of 0.66 and a phastCons100way_vertebrate of 0.93 (annotated by dbNSFPv3.5a) (21–23). The mutation is rare in the CNPRC colony (~0.95% allele frequency) and a detailed pedigree demonstrates a dominant inheritance pattern and facilitated discovery of *OPA1* homozygotes (Fig. 1).

### *OPA1* Homozygotes Have Findings Suggestive of RGC Loss and Dysfunction.

For all genotypes, intereye correlation was very strong for global RNFL thickness (R = 0.955, *P* = 9.5 × 10^−30^), supporting the use of eye-averaged values for subsequent analyses. In the two homozygous macaques identified (21.5-y-old female; 7.3-y-old male), while IOP was comparable to WT (*SI Appendix*, Fig. S1*A*) global circumpapillary RNFL thickness (*SI Appendix*, Fig. S1*B*), as well as PERG N35-P50 and P50-N95 amplitudes were reduced (*SI Appendix*, Fig. S1 *C* and *D*) relative to WTs; given the sample size of two, these observations are descriptive since no statistical comparisons were performed.

### *OPA1* Heterozygotes Have Structural Changes Consistent With RGC Loss.

We initially generated normative data from 113 wildtype (WT) rhesus macaques (0.33 to 29 y of age) with a mean ± SD (range) global peripapillary RNFL thickness of 108 ± 9 (85 to 131) μm consistent with previous studies in this species (24–26). Using linear regression analysis, global RNFL thickness progressively declined with age by 0.30 μm per year (*P* = 0.023) primarily due to a significant decrease in the nasal quadrant (*P =* 0.019, *SI Appendix*, Fig. S2). Females showed a significantly greater RNFL thickness (*P* = 0.043) in the superotemporal region (*SI Appendix*, Fig. S3). In the WT individuals, manual measurements differed significantly from automated ones in the inferior quadrant (*P* < 0.05, *SI Appendix*, Fig. S4*A*). Similarly, *OPA1* heterozygotes (n = 13) also displayed significant differences between the manual and automated measurements in the superotemporal region (*P* = 0.045), due to difficulties with the program identifying the RNFL (*SI Appendix*, Fig. S4*B*). As such, we present manual measurements since the software algorithm of the Heidelberg Spectralis is less accurate under diseased conditions (*SI Appendix*, Fig. S5 *A*–*C*) (25, 26).

**Fig. 2.**
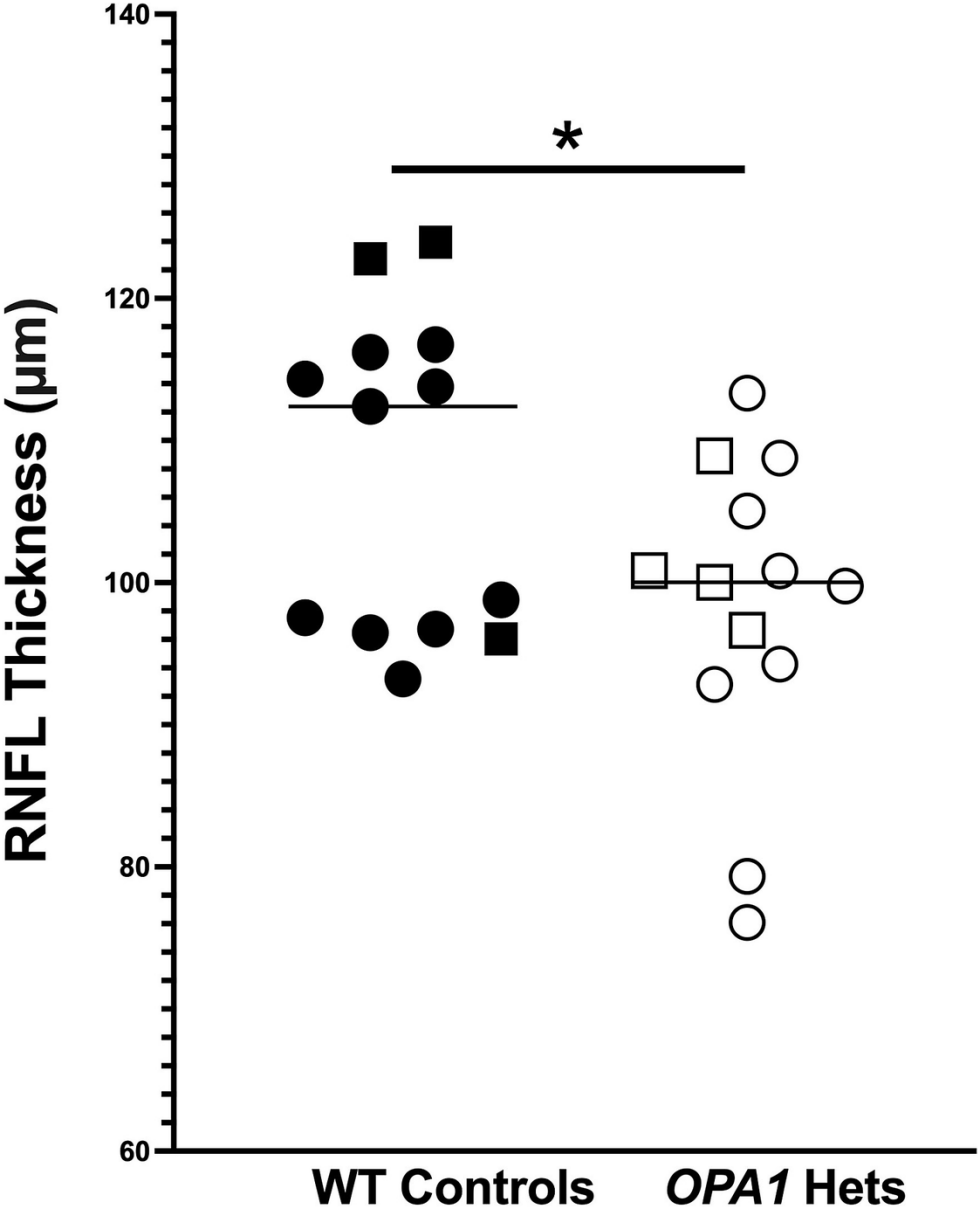
Rhesus macaques heterozygous for an *OPA1*A8S mutation had a thinner global circumpapillary retinal nerve fiber layer (RNFL) compared to their WT counterparts. Column scatterplot comparing manual global RNFL thickness in 13 *OPA1* heterozygotes [10.7 ± 6.9 (0.83 to 28) y; 9 females, 4 males] and 13 WT controls [10.5 ± 6.5 (0.58 to 24) y, 10 females, 3 males]; the RNFL thickness was manually measured and averaged from 24 segments surrounding the peripapillary ONH. For each macaque, measurements from each eye were averaged. Global peripapillary RNFL thickness was significantly lower in the *OPA1* heterozygotes at 96 ± 10 µm versus their age-, sex-matched WT controls at 108 ± 11 µm (*P =* 0.043). Males are represented by squares, and females by circles.

**Fig. 3.**
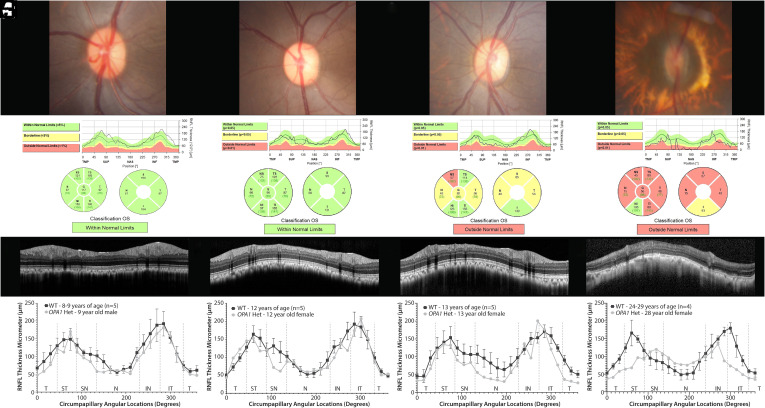
Rhesus macaques heterozygous for an *OPA1* mutation exhibit structural changes consistent with RGC loss of varying severity. The horizontal ovoid, pink optic nerve head (ONH) of a 9-y-old male macaque (*A*) differs from the pale ONHs, particularly temporally, observed in 12- and 13-y-old females (*B* and *C*) and the markedly smaller, atrophic ONH of a 28-y-old female (*D*; moderate cataract limited the quality of fundus photography in *D*). All macaques are heterozygous for the *OPA1*A8S mutation and left eyes are shown. Automated peripapillary scans of the 9-y-old male (*E*) and 12-y-old female (*F*) suggest fairly normal thickness in comparison to normal humans. The 13-y-old female demonstrates moderately decreased RNFL thickness with automated analysis that is most severe superonasal (*G*). In the 28-y-old female, most regions demonstrate markedly reduced RNFL thickness with automated scans (*H*). OCT b-scans in the same macaques further illustrate these findings (*I*–*L*); the cataract did not preclude acquisition of high quality ONH scans (*L*). Manual measurements in the 9-y-old *OPA1* heterozygote (*M*) and the 12-y-old *OPA1* heterozygote (*N*) demonstrate decreased RNFL thickness in the superonasal regions in comparison to mean ± SD measurements from age-matched WT macaques. The 13-y-old female demonstrates RNFL loss in most regions versus mean ± SD measurements from age-matched controls (*O*). In the 28-y-old female, manual measurements demonstrated a marked reduction in nearly every region in comparison to mean ± SD measurements from age-matched controls (*P*).

**Fig. 4.**
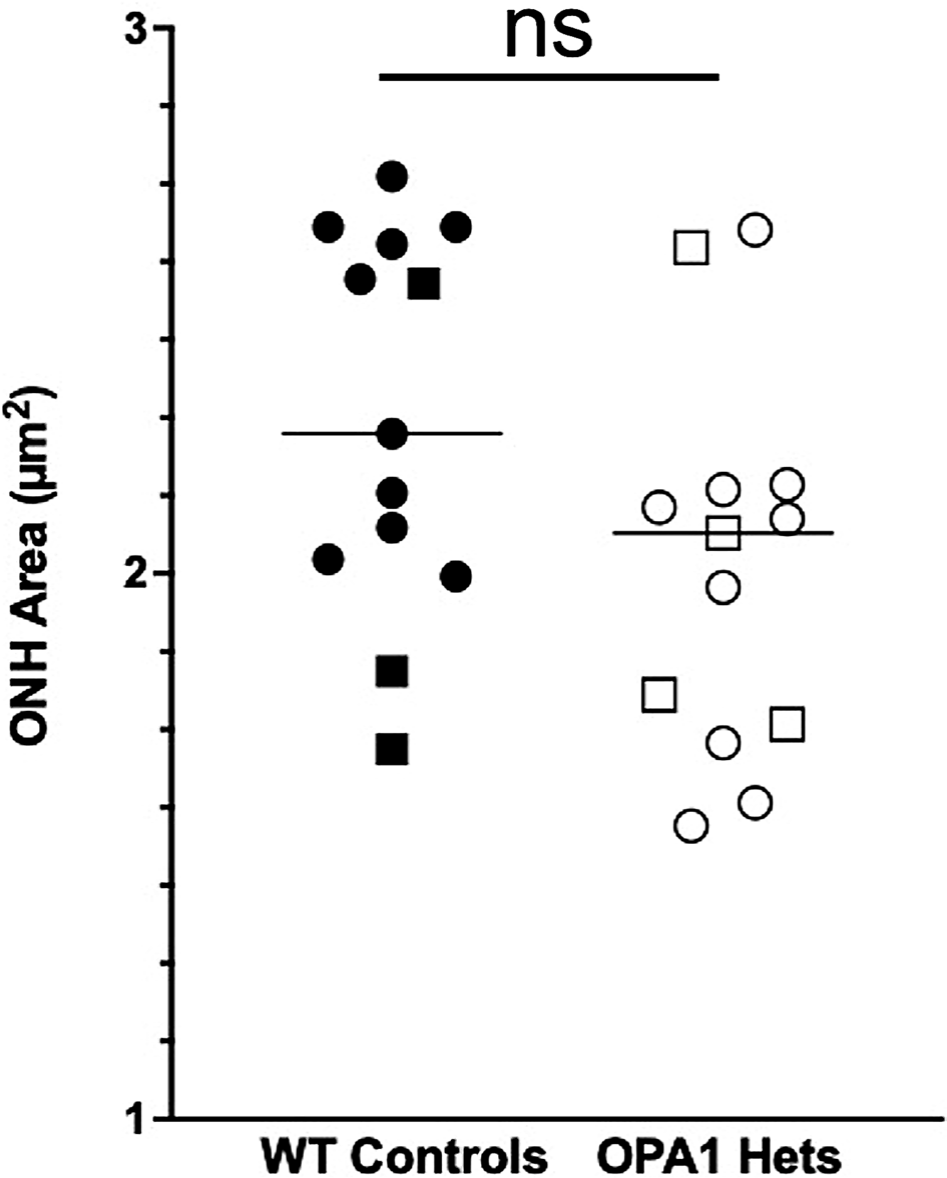
Optic nerve head (ONH) area did not significantly differ between *OPA1* heterozygotes and WT controls. Column scatterplot comparing manual optic disc area measurements between 13 *OPA1* heterozygotes [10.7 ± 6.9 (0.83 to 28) y; 9 females, 4 males] and 13 WT controls [10.5 ± 6.5 (0.58 to 24) y, 10 females, 3 males]. The optic disc area did not significantly differ in the *OPA1* heterozygotes at 2.04 ± 0.372 mm^2^ versus age-, sex-matched WT controls at 2.28 ± 0.35 mm^2^ (*P =* 0.099; ns, not significant). Males are represented by squares, and females by circles.

**Fig. 5.**
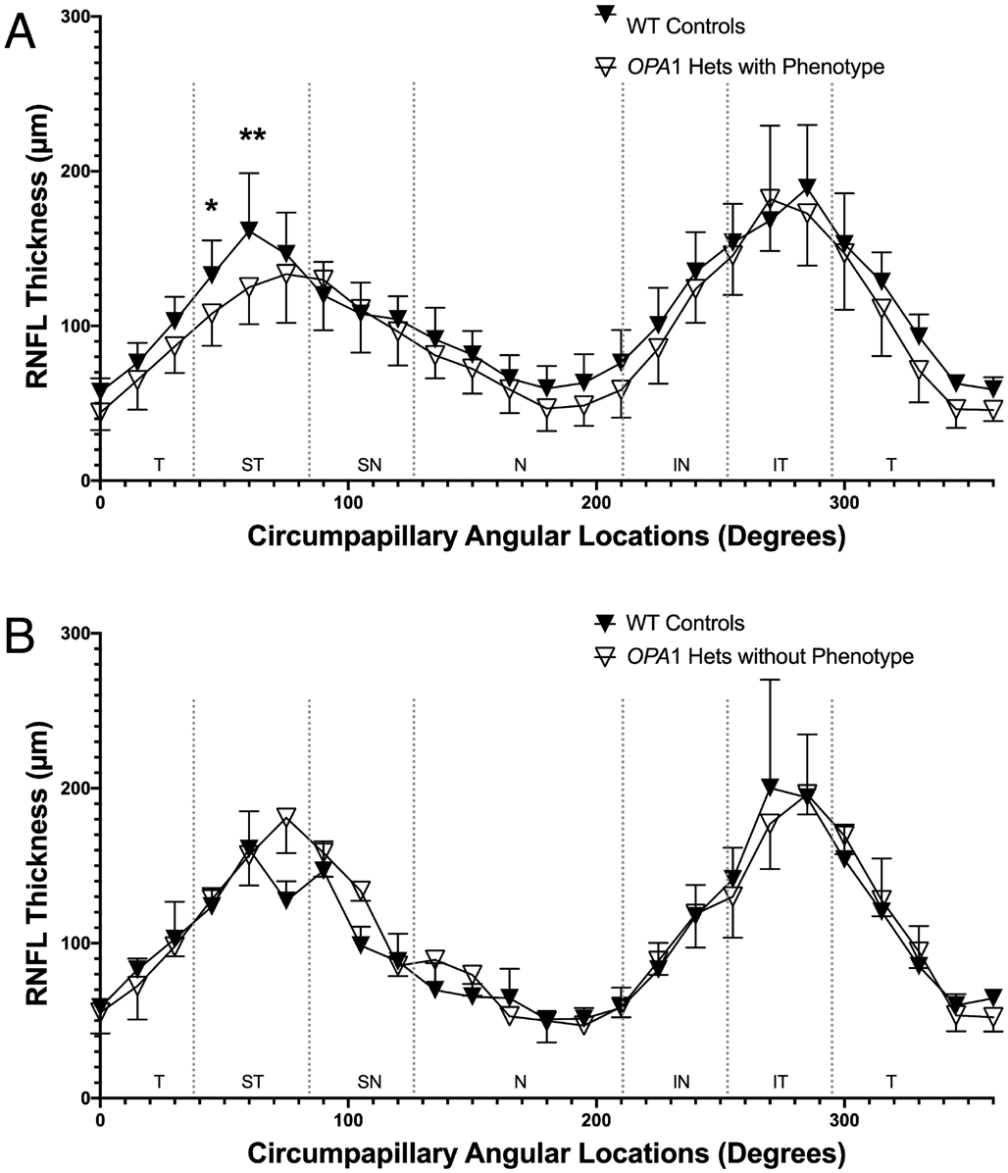
Ten *OPA1* heterozygotes with a phenotype had a significantly thinner RNFL in the superotemporal region versus WT controls, while three *OPA1* heterozygotes without a phenotype did not have a significant difference in RNFL thickness in any region. (*A*) Phenotypic changes were defined as a change in RNFL thickness observed in the *OPA1* heterozygotes beyond the SD of 5 WT controls in at least one region of the peripapillary RNFL. In the 10 *OPA1* heterozygotes with a phenotype [12.6 ± 6.2 (4.6 to 28) y; 7 females, 3 males], there were two superotemporal regions that were significantly thinner in comparison to age-, sex-matched WT controls (45° *P* = 0.0209, 60° *P* = 0.0007). (*B*) By contrast, the 3 *OPA1* heterozygotes without a phenotype [4.4 ± 6.3 (0.11 to 14) y, 2 females, 1 male] did not display a significantly different RNFL thickness in any region in comparison to age-, sex-matched WT controls (*P* > 0.05).

Given the impact of sex and age on RNFL thickness of the WT individuals, we compared the *OPA1* heterozygotes with age-, sex-matched WT controls. Specifically, 13 *OPA1* heterozygotes (9 females, 4 males) 10.7 ± 6.9 (range 0.83 to 28) years of age (y) and 13 WT controls (10 females, 3 males) 10.5 ± 6.5 (0.58 to 24) y, were assessed. Global peripapillary RNFL thickness was significantly lower in the *OPA1* heterozygotes at 96 ± 10 µm versus age-, sex-matched WT controls at 108 ± 11 µm (*P =* 0.043, Fig. 2). When assessing individuals, we noted a spectrum of different phenotypes for RNFL thickness (Fig. 3 and *SI Appendix*, Fig. S6) and optic nerve head (ONH) area (Fig. 4). We individually compared each *OPA1* heterozygote with 5 WT controls and determined that three did not display a phenotype as defined by decreased RNFL thickness in at least one region (*SI Appendix*, Fig. S6 *K*–*M*). The temporal (n = 7), nasal (n = 7), and superotemporal (n = 5) regions were most commonly reduced while thinning was less commonly observed in the inferotemporal (n = 3), superonasal (n = 3), and inferonasal (n = 2) regions. In the 10 *OPA1* heterozygotes with a RNFL thinning phenotype, there were two superotemporal regions that were significantly thinner in comparison to age-, sex-matched WT controls (45° *P* = 0.0209, 60° *P* = 0.0007, Fig. 5*A*). As expected, the 3 *OPA1* heterozygotes without a phenotype did not demonstrate a significantly different RNFL in any region in comparison to age-matched WT controls (*P* > 0.05, Fig. 5*B*). We highlight that the 10 *OPA1* heterozygotes with RNFL thinning were significantly older than the 3 without one at 12.6 ± 6.21 and 4.4 ± 6.3 y, respectively (*P =* 0.0385).

The optic disc area of 109 WT individuals was 2.06 ± 0.55 (1.40 to 2.99) mm. No significant difference was observed with age (*P* = 0.089, *SI Appendix*, Fig. S7*A*). Additionally, optic disc area did not significantly differ between age-matched WT males and females (*P* = 0.44, *SI Appendix*, Fig. S7*B*). While optic disc area did not significantly differ between the *OPA1* heterozygotes and their age-, sex-matched WT controls (*P =* 0.099, Fig. 4), we did identify one 28-y-old female *OPA1* heterozygote with marked ONH atrophy and reduction in optic disc area (Fig. 3*D*). Seven of the 13 *OPA1* heterozygotes (58%, 9- to 28-y-old) had temporal ONH pallor (Fig. 3 *B* and *C*). Intraocular pressure (IOP) did not significantly differ (*P* = 0.303) between WT and *OPA1* heterozygotes at 15 ± 4 and 16 ± 4 mm Hg, respectively.

Macular thickness maps were acquired for the RNFL, GCL, and IPL, and these were combined to generate ganglion cell complex (GCC) thickness and showed trends in qualitatively reduced thickness in *OPA1* heterozygotes compared with WT controls (*SI Appendix*, Figs. S8 and S9).

### *OPA1* Heterozygotes Show Functional Deficits On Pattern Electroretinography (PERG) and Visual Behavioral Tasks But Do Not Display Hearing Abnormalities.

**Fig. 6.**
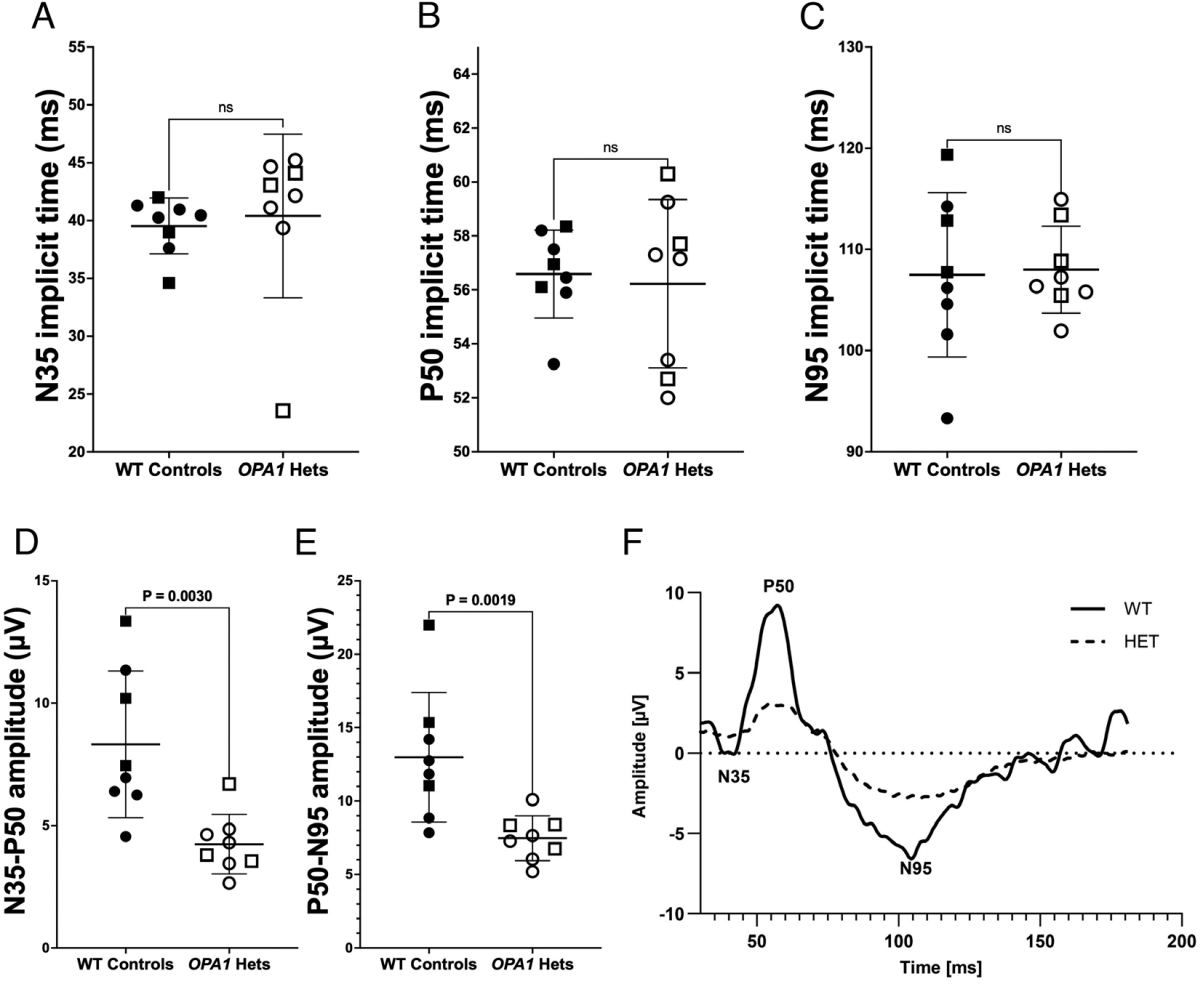
Reduced RGC function was observed with PERG in *OPA1* heterozygotes. In 8 WT versus 8 *OPA1* heterozygous rhesus macaques, implicit times did not significantly differ for the N35 (*A*, 39.5 ± 3.56 and 40.4 ± 7.12 ms), P50 (*B*, 56.6 ± 1.83 and 56.2 ± 3.14 ms), and N95 (*C*, 108 ± 8.40 and 109 ± 5.77 ms) components (all *P >* 0.05, *A*–*C*). However, amplitudes of N35-P50 and P50-N95 were significantly reduced in *OPA1* heterozygotes versus WT controls at 4.24 ± 1.48 versus 8.31 ± 3.18 µV (*D*, *P* = 0.0030) and 7.47 ± 1.74 µV versus 13.0 ± 4.06 µV (*E*, *P* = 0.0019), respectively. A representative waveform from an 11-y-old male *OPA1* heterozygote displays decreased N35-P50 and P50-N95 amplitudes in comparison to an age-, sex-matched control (*F*). Males are represented by squares, and females by circles.

The PERG P50-N95 amplitude showed very strong intereye agreement (R = 0.961, *P* = 0.00030), supporting the use of eye-averaged values for subsequent analyses. Implicit times for the N35, P50, and N95 components of the PERG were 39.5 ± 3.56, 56.6 ± 1.83, and 108 ± 8.40 ms, respectively, in age-matched and sex-matched WT controls and did not significantly differ from that of *OPA1* heterozygotes at 40.4 ± 7.12, 56.2 ± 3.14 and 109 ± 5.77 ms, respectively (Fig. 6 *A*–*C*). The mean amplitudes of N35-P50 and P50-N95 components were significantly lower in *OPA1* heterozygotes (4.24 ± 1.48 and 7.47 ± 1.74 µV; *P* = 0.0030) versus WT controls (8.31 ± 3.18 and 13.0 ± 4.06 µV; *P* = 0.0019; Fig. 6 *D*–*F*).

Two *OPA1* heterozygous macaques (18.5-y-old male and 20-y-old female) tested with visual behavioral task looking durations, which are associated with better vision, were in the lower third of a similarly aged cohort, with the female in the lowest quartile (*SI Appendix*, Data 1). Across the full sample of rhesus macaques (males and females, 4 to 21 y), mean looking time was 24.38 s (N = 18, SD = 2.52). For the *OPA1* heterozygous male, mean looking time on a different test declined from 20.47 s at 8.3 y to 8.15 s at 19.5 y, whereas looking times for the other macaques remained stable across sessions. Global mean RNFL thickness was 82 µm in the female at 21 y and 84 µm in the male at 20.1 y. In comparison, WT rhesus macaques 20 to 21-y-old from our normative cohort of 113 animals had a mean ± SD global peripapillary RNFL thickness of 98.97 ± 6.38 µm, confirming lower RNFL values in the *OPA1* heterozygotes than in age-matched controls.

In a subset of five *OPA1* heterozygotes, brainstem auditory evoked responses (BAER) were within normal limits in both ears, and no ataxia or other neurologic abnormalities were observed (*SI Appendix*, Fig. S10).

### Histopathologic Evidence of Progressive RGC and Optic Nerve Degeneration in *OPA1* Heterozygotes.

**Fig. 7.**
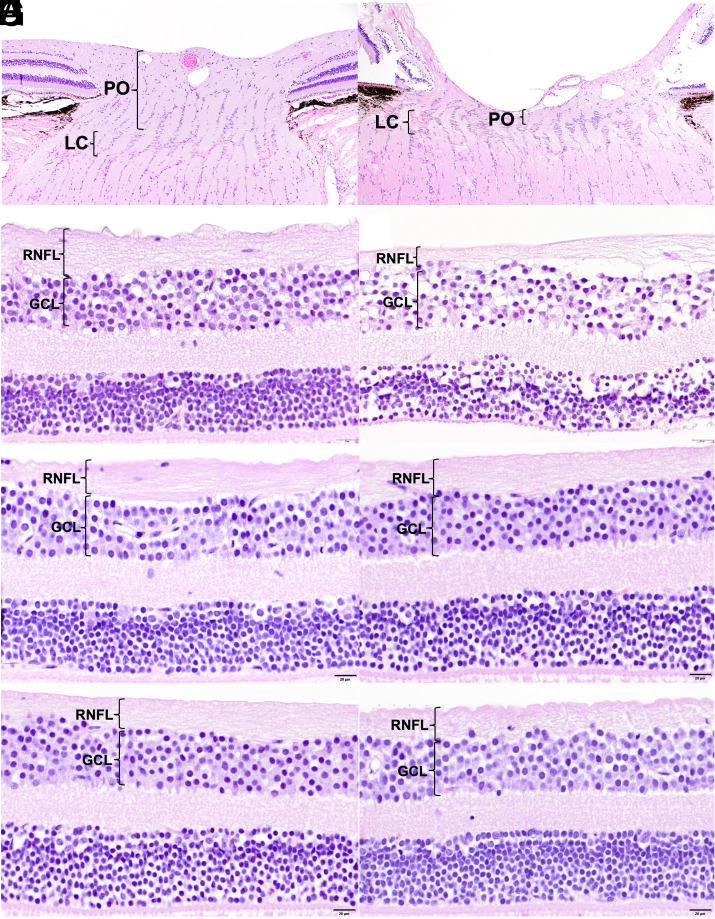
Marked loss of prelaminar optic nerve tissue (PO), RNFL and ganglion cell layer (GCL) is observed in a 17.2-y-old *OPA1* heterozygote in comparison to an age-matched control rhesus macaque. Microscopic images of the optic nerve from a 16.9-y-old WT control (*A*) and 17.2-y-old *OPA1* mutant heterozygote (*B*). Additional central peripapillary retinal images from these two macaques (*C*, *D*) illustrate the loss of GCL somas and axons in the *OPA1* heterozygote (*C*). Normal retinal architecture is observed in an 8.3-y-old WT (*E*) and an 8.4-y-old *OPA1* heterozygote (*F*). A 4.5-y-old WT also has normal retinal structure (*G*) and a 6.4-y-old *OPA1* heterozygote (*H*) demonstrates no major changes at this age. LC=lamina cribrosa. H&E stain.

Three male *OPA1* heterozygotes aged 6.4, 8.4, and 17.2 y underwent histological assessment and were compared with WT controls, a 4.5-y-old female, 8.3-y-old male, and 16.9-y-old male. No obvious differences in optic nerve morphology, RNFL thickness, and ganglion cell density were observed between the 6 and 8-y-old *OPA1* heterozygotes and their age-matched individuals (Fig. 7). When compared to its age-matched control and the two younger affected macaques, the 17.2-y-old *OPA1* heterozygote demonstrated moderate ganglion cell nuclear and axonal loss and thinning of the RNFL at the central peripapillary retina along with diffuse atrophy of the ONH, characterized by significant thinning of the prelaminar neuropil (ganglion cell axonal bundles) (Fig. 7).

**Fig. 8.**
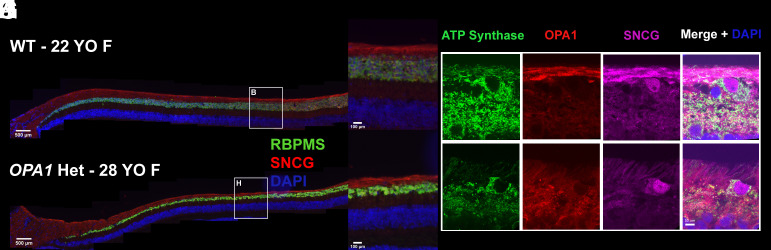
Retinal ganglion cells (RGCs) and RNFL are appropriate in a 22-y-old WT control (*A*–*F*) and markedly reduced with absent OPA1 from the RNFL and decreased mitochondria in an *OPA1* heterozygote (*G*–*L*). The white box in A indicates the perifovea and correspond to magnified section in B; this location was confirmed using antibodies to rod and cone photoreceptors. Sections of peripheral temporal retina demonstrate a high density of mitochondria (ATP synthase, green) in axons and soma (*C*) but OPA1 (red) primarily localized to RGC axons (SNCG, purple) in the 22-y-old WT control (*D*–*F*). The white box in G indicates the perifovea and correspond to magnified section in H. By comparison, in the 28-y-old *OPA1* heterozygote there was dramatically decreased mitochondria density in the IPL (*I*) with no OPA1 present in the RNFL (*J–L*). The RGC soma and axons were labeled with RBPMS (green) and SNCG (red), respectively; nuclei were labeled with DAPI (blue).

To analyze how the *OPA1A8S* mutation affects OPA1 localization, RGC and RNFL morphology, and mitochondrial impact in RGCs, histological samples were collected in a 22-y-old female WT control (Fig. 8 *A*–*F*) and a 28-y-old female rhesus macaque *OPA1* heterozygote (Fig. 8 *G*–*L*). Retinal sections showed marked loss of RGCs (>50%) with RPBMS and extensive RNFL thinning with SNCG in the age-, sex-matched WT control (Fig. 8 *A* and *B*) *OPA1* heterozygote (Fig. 8 *G* and *H*) versus the age-, sex-matched WT control while the outer retina remained preserved. Retinal sections of the peripheral temporal retina were used to determine mitochondrial impact using ATP synthase and OPA1 localization. In the WT control, there was high mitochondrial density in the RNFL axons and RGC soma with OPA1 localizing primarily in the RNFL axons, with low levels of OPA1 present in the synaptic layers compared to other cells (Fig. 8 *C*–*F*). By contrast, the *OPA1* heterozygote has dramatically decreased mitochondrial density in the inner plexiform layer and RNFL axons, with markedly reduced OPA1 in the RNFL axons (Fig. 8 *I*–*L*).

**Fig. 9.**
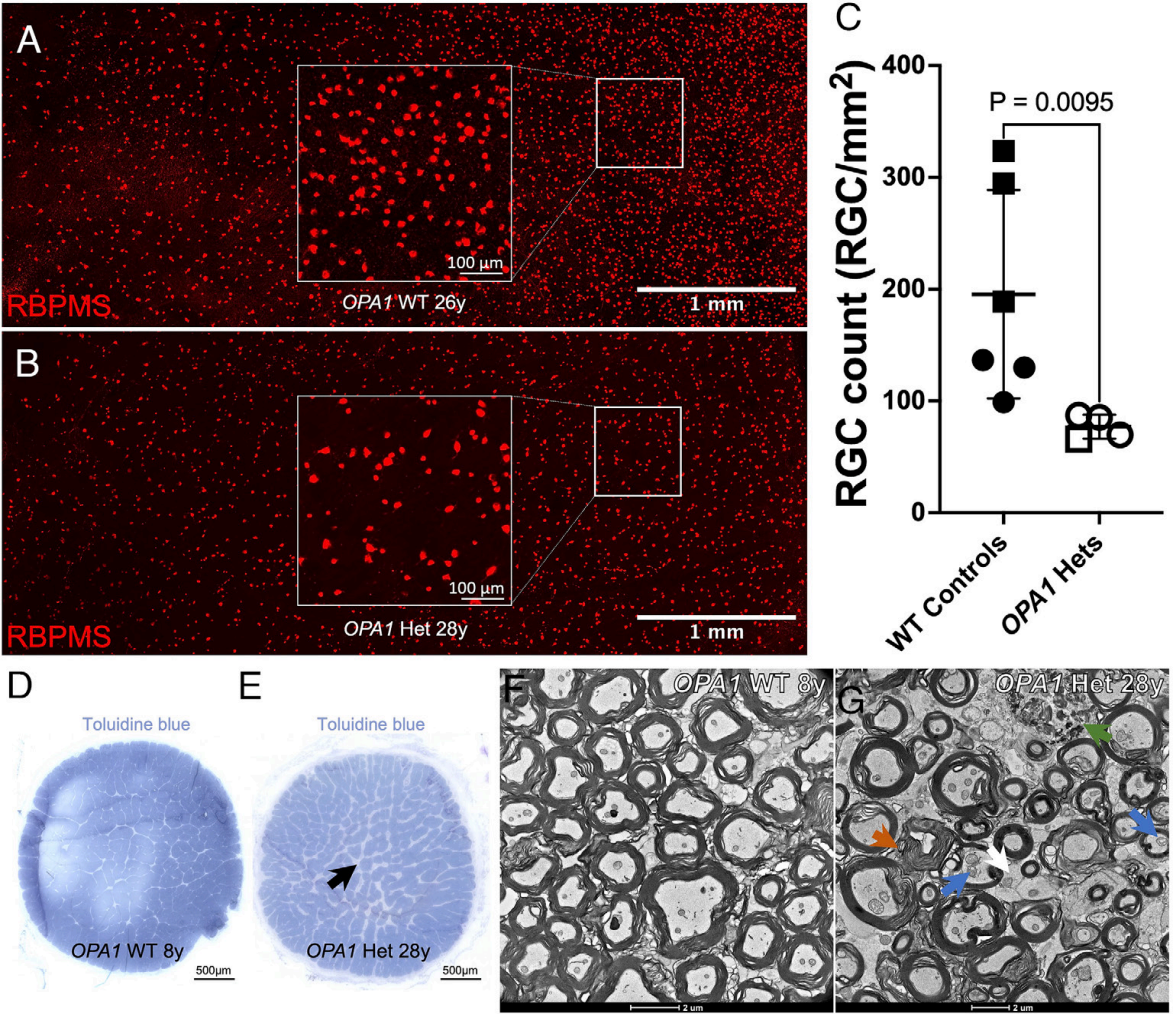
Decreased RGC density in *OPA1* heterozygotes compared to WT controls. (*A*) Representative RBPMS-immunolabeled temporal retina periphery wholemount from a 26-y-old WT control showing a dense RGC mosaic; left edge of the image represents the temporal-most region of retina containing RGCs. (*B*) Same area imaged from a 28-y-old *OPA1* heterozygous macaque, illustrating reduced RGC density at all eccentricities. Insets show higher magnification of regions approximately 4 mm from the temporal retina margin. (*C*) Quantification of RGC density (RGC/mm^2^) in WT controls (n = 6, average age 17.0 y) and *OPA1* heterozygotes (n = 4, average age 16.6 y). Each data point represents the mean RGC count from three nonoverlapping fields acquired 4 mm from the temporal retinal margin in a single eye. Data are presented as mean ± SD; *OPA1* heterozygotes show a significant reduction in RGC density compared to controls (*P* = 0.0095). Males are represented by squares, and females by circles. (*D* and *E*) Toluidine blue-stained semithin cross-sections of optic nerve from an 8-y-old WT macaque (*D*) and a 28-y-old *OPA1* heterozygous macaque (*E*) showing diffuse rarefaction of myelinated fibers and widening of interaxonal spaces compared to the control, consistent with axon loss (black arrow). (*F* and *G*) Transmission electron micrographs obtained from central regions of the optic nerve from an WT macaque (*F*, 8 y) and an *OPA1* heterozygous macaque (*G*, 28 y). The *OPA1* heterozygote shows swollen and dysmorphic mitochondria (blue arrows), distended and irregular myelin sheaths (orange arrow), and accumulation of electron-dense debris (green arrow) as well as hypertrophic/distended astrocytic processes between axons (white arrow).

Quantitative analysis of RBPMS-labeled retinal wholemounts demonstrated a marked loss of RGC density in *OPA1* heterozygous macaques compared to WT controls (Fig. 9 *A*–*C*; *P* = 0.0095; n = 4 *OPA1* Het, n = 6 WT). On toluidine blue-stained semithin cross-sections of optic nerve, *OPA1* heterozygous macaques showed diffuse rarefaction of myelinated fibers and widening of interaxonal spaces compared with WT controls, consistent with axon loss (Fig. 9 *D*–*E*, black arrow). With TEM of the same cohort, tightly packed myelinated axons with relatively uniform myelin sheaths and preserved mitochondrial morphology in WT nerves were observed (Fig. 9*F*). By contrast, *OPA1* heterozygous nerves exhibited swollen and dysmorphic mitochondria (Fig. 9*G*, blue arrows), distended and irregular myelin sheaths (Fig. 9*G*, orange arrow), and accumulation of electron-dense debris (Fig. 9*G*, green arrow) as well as hypertrophic/distended astrocytic processes between axons (Fig. 9*G*, white arrow).

## Discussion

The present work establishes a large-animal animal model for human ADOA – the rhesus macaque (*Macaca mulatta).* In humans, ADOA is an inherited optic neuropathy with an estimated incidence of 1:12,000 to 1:50,000 that results in slowly progressive vision impairment and has no effective treatment (9, 27). Mutations in *OPA1*, which encodes a mitochondrial dynamin-related protein critical for mitochondrial function, are responsible for most ADOA cases (28). Thus, *OPA1* mutations result in abnormal mitochondrial metabolism, impaired oxidative phosphorylation, and increased ROS concentrations ultimately leading to apoptosis of RGCs (9, 21). Given the prominent papillomacular pathology observed in humans with ADOA (28), a foveate species similar to humans would provide an optimal model for studying its etiopathogenesis and potential treatments. Herein, we present this novel NHP model of ADOA. We highlight the use of a targeted sequencing approach combined with an updated rhesus macaque gene assembly to identify putative retinal or neurological disease-causing mutations (23, 29). Indeed, several spontaneous retinal diseases with a known genotype have been recently identified in rhesus macaques including Batten disease, achromatopsia, Bardet–Biedl syndrome, and oculocutaneous albinism (30–34). In the present study, this targeted sequencing approach revealed the presence of a missense mutation [NM_015560.2:c.22G>T(p.ala8ser)] in *OPA1* in the mitochondrial targeting sequence of exon 1 in rhesus macaques from CNPRC (23). The analogous mutation in humans has previously been reported in ADOA patients (20). Furthermore, the mutation was rare in human and rhesus macaque populations, further supporting its pathogenic role in ADOA (23). Finally, a single NHP model of ADOA in a less variable environmental background than in humans enables the study of phenotypic variability associated with *OPA1A8S* heterozygosity and eventually homozygosity.

We demonstrated a spectrum of phenotypes in rhesus macaques heterozygous for this *OPA1* mutation ranging from absent to severe consistent with the human condition (4). As is typical in human cases, the clinical fundus appearance was relatively normal in most *OPA1* heterozygotes with mild, temporal ONH pallor noted in 58% individuals and marked ONH atrophy in the oldest individual (28 y) with the most severe phenotype. In ~70% of individuals with a phenotype, peripapillary and papillomacular RNFL loss was documented with spectral-domain optical coherence tomography (SD-OCT) and histopathology, respectively, congruous with ADOA (10, 35). Specifically, the superotemporal and temporal regions of the peripapillary RNFL, which thin first in patients, were the most common regions to be decreased in the *OPA1* heterozygotes (36). Macular GCC thickness maps likewise showed a trend toward thinner GCC in *OPA1* heterozygotes particularly in the central region. Furthermore, RGC dysfunction was identified in all *OPA1* heterozygotes tested with PERG mimicking what is observed in ADOA patients (2, 3). In humans, approximately 10 to 20% of individuals with ADOA develop additional neurological manifestations such as sensorineural hearing loss and ataxia (37). None of our *OPA1* heterozygous macaques shows ataxia or other neurological deficits, and BAER in five macaques confirmed normal hearing bilaterally. To date, no features of an ADOA plus phenotype have been identified in this cohort.

In contrast to rodent *Opa1* knockouts, which are embryonically lethal, and the rare human cases of biallelic *OPA1* mutations that present with severe multisystem disease and early neonatal death, homozygous *OPA1* macaques in our colony survive into adulthood and are fertile (38, 39). Characterization of these macaques is ongoing and suggestive of an ADOA-like phenotype similar to heterozygotes. The survival of these homozygotes without systemic disease may reflect a hypomorphic effect of the variant, which does not involve a canonical critical exon and therefore may preserve partial protein function. Species-specific differences, including genetic background and redundancy within mitochondrial pathways, may also contribute to the absence of lethality (40). In this context, RGCs are particularly sensitive to impaired mitochondrial function, and preferential vulnerability of the optic pathway is well documented in mitochondrial optic neuropathies, which provides a plausible explanation for an ocular phenotype in the absence of systemic disease (41, 42).

To understand the impact of this *OPA1* mutation on RGC morphology and mitochondrial function, we performed histology, immunohistochemistry, and TEM on retinal sections from *OPA1* heterozygotes and WT controls. The *OPA1* heterozygotes showed a clear reduction in RGC density and RNFL thinning together with widespread ultrastructural abnormalities on TEM, including dysmorphic mitochondria, axon loss, myelin disruption, and hypertrophic astrocytic processes. Thus, these findings demonstrating RGC loss and mitochondrial pathology central to the pathogenesis of *OPA1-*associated ADOA further support the utility of this NHP model in studying the pathology of this disease, particularly since histological and immunohistochemical studies of human eyes are limited from prolonged death-to-preservation time, cell death from transection of RGC axons during eye removal, and unreliable ascertainment of disease phenotype due to variable quality of donor records (27).

Finally, we provide normative peripapillary RNFL thickness data in a large cohort of rhesus macaques WT for this *OPA1* mutation spanning 4 mo to 29 y. These observations underscore the importance of comparing *OPA1* heterozygotes with age-, sex-matched WT controls in this rhesus macaque population and likely in human clinical trials evaluating the natural history of ADOA. We also observed significant differences between automated and manual measurements of the peripapillary RNFL in both WT and *OPA1* heterozygous macaques. In particular, automated measurements showed segmentation errors in some RNFL regions of the *OPA1* heterozygotes, emphasizing the importance of manual measurements in diseased conditions (43) and presumably in human clinical trials evaluating novel diagnostic tools or therapeutic strategies for ADOA. RNFL thickness was modestly lower in *OPA1* heterozygotes in comparison to age-, sex-matched WT controls; however, given substantial variability in both groups, it should be interpreted as one element within a multifactorial phenotype that also includes electrophysiologic dysfunction, optic nerve head changes, and RGC pathology.

These *OPA1* heterozygous rhesus macaques offer a compelling model to investigate novel therapies for ADOA, including neuroprotective strategies relevant to other heritable optic neuropathies as well as those caused by glaucoma, trauma, and inflammation (19, 28). Indeed, mitochondrial dysfunction is central to the pathogenesis of the aforementioned conditions, and neuroenhancement therapies with efficacy in this ADOA NHP model are likely to translate more effectively to humans than those only evaluated in mice. A range of therapies for ADOA including neuroprotective compounds and gene augmentation have been evaluated in mouse models (44, 45). However, when transitioning to human clinical trials, only a limited number have been conducted, primarily focusing on idebenone and specific gene therapy interventions targeting *OPA1* mutations (28). To date, the only ADOA human clinical trial to demonstrate efficacy was for a small, short-term study of idebenone (12). Promising gene therapy approaches, including gene augmentation utilizing adeno-associated viral vectors and mRNA strategies such as antisense oligonucleotides have demonstrated potential in preclinical studies but may benefit from study in this NHP model before proceeding to clinical trials (46–49).

In summary, our study demonstrates the utility of an NHP model with the *OPA1A8S* mutation for investigating the pathogenesis and phenotypic variability of ADOA. The NHP model closely recapitulates the human papillomacular pathology observed in ADOA patients. In addition, our findings provide important insights into the role of *OPA1* mutations in mitochondrial dysfunction, RGC loss, and optic nerve degeneration. Finally, this model can serve as a valuable tool for further understanding the mechanisms underlying ADOA, developing novel diagnostic tools for this condition, and exploring potential therapeutic interventions.

## Materials and Methods

### Animals.

The rhesus macaques (*Macaca mulatta*) were examined live at the CNPRC at the University of California, Davis (UC Davis), an institution accredited by the Association for Assessment and Accreditation of Laboratory Animal Care (AAALAC) International. This study adhered to all guidelines from the Association for Research in Vision and Ophthalmology Statement for the Use of Animals in Ophthalmic and Vision Research and was performed in accordance with the NIH Guide for the Care and Use of Laboratory Animals. We performed blood collection, ophthalmic examinations, and phenotyping according to a protocol approved by the Institutional Animal Care and Use Committee at UC Davis. Venous blood was collected, and DNA was extracted using routine methods and DNA preserved at −80 °C until shipment to Baylor College of Medicine.

### Sex as a Biological Variable.

Both male and female rhesus macaques were included in the present study. Sex differences were determined for global RNFL thickness in WT rhesus macaques.

### Genetic Sequencing and Analysis.

Targeted sequencing (TSC) that captures the coding regions of 286 inherited retinal disease genes and whole exome sequencing (WES) were performed on 685 rhesus macaques according to the protocol from the manufacturer (N = 65 for TSC only, N = 564 for WES only, and N = 56 for both TSC and WES). Briefly, Covaris was used to shear 1 ug genomic DNA for 70 s and Ampure XP beads were used for the purification. Following end repair and A-tailing, the product was added with indexed adaptors. Ampure XP beads were used to purify the product, and KAPA Hifi HotStart ready mix was used for amplification (KAPA HyperPrep Kit, Roche).

For targeted capture sequencing, 40 to 50 libraries were pooled for Agilent SureSelect Target enrichment system following the manufacturer’s protocol with modification. Briefly, pooled samples were hybridized to probe pool, and captured with Dyanbeads MyOne Streptavidin T1 magnetic beads. After wash, captured DNA was amplified with Phusion High Fidelity DNA polymerase (NEB). After cleanup and quantification, the Illumina Novaseq6000 Sequencer was used to sequence the diluted library.

For WES, 10 to 12 libraries were pooled for Nimblegen SeqCap EZ protocol (Roche) using SeqCapEZ Choice probe with Monkey spike in. Briefly, pooled samples were hybridized to probe pool, and captured with Dyanbeads M270 Streptavidin. After wash, captured DNA was amplified with KAPA HIfi Hotstart Readymix (KAPA). After cleanup and quantification, the Illumina Novaseq6000 Sequencer was used to sequence the diluted library.

BWA mem was used to align the sequencing reads to the rhesus reference genome assembly (Mmul_8.0.1 or Mmul_10). Following the GATK pipeline, the single nucleotide variants (SNVs) and short insertion/deletions (indels) were called. The variants were annotated with variant effect predictor (VEP) based on merged Ensembl and RefSeq gene models of Mmul_8.0.1 or Mmul_10. The variants in rhesus genome position were then lifted over to the orthologous human position. The protein-altering effects of the variants in human coordinate were annotated with ANNOVAR (v. 07/17/2017) (50) and dbNSFP (v.3.5a, includes SIFT, PolyPhen-2, etc.) (21, 22) based on the gene model of hg19. The pathogenic variant was identified by screening the variants in the Human Gene Mutation Database (HGMD). Additional rhesus candidates with the *OPA1* mutation were confirmed by direct PCR Sanger sequencing.

### Ophthalmic Phenotyping.

Phenotypic data were collected from WT rhesus macaques either prior to use in a study or from a screening program to identify NHPs with age-related or potentially inherited ocular abnormalities; 6 were included in a previously published study (51). Sedation, ocular examinations, intraocular pressure measurements, fundus photography, and SD-OCT with confocal scanning laser ophthalmoscopy (cSLO) were performed as previously described (51).

While behavioral visual data were not available on the macaques included in the physiological and histological analyses, we had two *OPA1* heterozygotes enrolled on long-term projects including visual tasks. For more details see *SI Appendix*, Data 1.

### Optical Coherence Tomography (OCT) and Retinal Layer Measurements.

Confocal scanning laser ophthalmoscopy with SD-OCT was performed with a Spectralis® HRA + OCT (Heidelberg, Germany). The standard glaucoma setting of the Heidelberg Spectralis HRA+OCT platform was used to measure RNFL thickness (*SI Appendix*, Fig. S1). Images and measurements of the peripapillary RNFL were obtained using a standard 12-degree diameter circular B-scan, with 1536 A-scans, centered on the ONH; three scans will be acquired per eye. Automated reports were generated from the Spectralis® HRA + OCT program which provided 768 data points of the RNFL across the circumpapillary retina. From the dataset, 25 points that corresponded to each 15-degree segment around the circumpapillary area for further analysis. Retinal layer segmentation was performed by a single experienced grader (TN) with the ImageJ software tool (NIH, Bethesda, MD). Additionally, macular volume scans were acquired on the Spectralis OCT (193 B-scans) to generate layer thickness maps for RNFL, GCL, and IPL. For each eye, parafoveal and perifoveal values were calculated as the mean of the nasal, temporal, superior, and inferior quadrants. Ganglion cell complex (GCC) thickness was computed per location (central, parafoveal, perifoveal) as RNFL + GCL + IPL. Group differences between WT controls and *OPA1* heterozygotes were tested with two-sided Wilcoxon tests for each outcome and location.

### PERG.

Commercial ERG equipment (RETIport 32; Roland Instruments, Brandenburg, Germany) that conformed to the 2012 International Society of Clinical Electrophysiology and Vision guidelines was used to record the PERG readings. The reference electrode was placed on the skin near the lateral canthus of eye being tested and the ground electrode was placed on the forehead. Eight WT controls (3 males and 5 females, 7 to 14 y of age) and 8 *OPA1* heterozygotes (3 males and 5 females, 7 to 14 y of age) were evaluated. The PERG was recorded using a black and white checkerboard stimulus with 97% contrast and mean luminance of 80 cd/m^2^. The stimulus frequency was 4.28 reversals/sec. The field size was 52° with a check size of 48 min. Band pass filter was set to 5 to 100 Hz with a sampling rate of 2.84 kHz. Artifact reject threshold was above 85%. The implicit times of N35, P50, and N95 were recorded, and the amplitudes of N35-P50 and P50-N95 were also measured.

### Histopathology.

Ocular tissues from three *OPA1* heterozygote males (6.4, 8.4, and 17.2 y old) and three age-matched WT control (4.5-y-old female, 8.3- and 16.9-y-old males) rhesus macaques were examined. Tissues were fixed in 10% buffer formalin phosphate (Fisher Scientific, Warehouse CA) for over 48 h and processed through an Epredia™ Excelsior™ AS tissue processor following the manufacturer-suggested protocol. The processed tissues were embedded in paraffin, and 4 µm paraffin sections were made using a Microm HM355S microtome. Slides were stained using an Epredia™ Gemini™ AS automated slide stainer (Fisher Scientific, Warehouse CA) following manufacturer-suggested Hematoxylin and Eosin (HE) stain protocol. Richard-Allan Scientific hematoxylin 7211, clarifier 1, bluing reagent, and eosin-Y alcoholic (Fisher Scientific, Warehouse CA) were used for the HE stains.

### Immunohistochemistry.

Ocular tissues from a 28-y-old female *OPA1* heterozygote with clinical phenotyping (Fig. 3) and a 22-y-old female WT rhesus macaque were recovered immediately following humane euthanasia for reasons unrelated to the study, in accordance with IACUC approved protocols and AVMA guidelines. Within 1 h of euthanasia, eyes were fixed in 4% paraformaldehyde. The retinas were removed and cryoprotected by overnight incubation with 30% sucrose solution prior to three consecutive freeze-thaw cycles in 30% sucrose before immunolabeling. The retinas were rehydrated three times (10 min each) in 1x phosphate-buffered saline (PBS) with 0.1% Triton X 100 (Sigma-Aldrich, St. Louis, MO) and 0.1% bovine serum albumin (BSA) (Sigma-Aldrich, St. Louis, MO). Retinas were then blocked for 1 h in either 10% donkey serum or 10% goat serum (Jackson ImmunoResearch, West Grove, PA). Tissues were then incubated overnight at 4 °C in block solution with primary antibodies against Mouse-anti-ATP Synthase (1:500; 05-709, Upstate, Darmstadt, DE); Goat-anti-OPA1 (1:200; Sc-30572, Santa Cruz Biotechnology, Santa Cruz, CA); Mouse-anti-SNCG (1:500; 2C3, WH00006623M1, Sigma-Aldrich, St. Louis, MO); Rabbit-anti-SNCG (1:1,000; Custom generated to C-terminal 16 aa of mouse SNCG by Covance, Princeton, NJ) (59); Anti-Cone Arrestin (1:250; EMD Millipore, Burlington, MA); Anti-Mammalian Rhodopsin monoclonal antibody 2B2 (1:25; courtesy of Robert S. Molday) or Rabbit-anti-RBPMS (1:1,000; GTX118619, Genetex, Irvine, CA). The retinal sections were then rinsed three times (10 min each) in 1x phosphate-buffered saline (PBS) with 0.1% Triton X 100 (Sigma-Aldrich, St. Louis, MO) and 0.1% bovine serum albumin (BSA) (Sigma-Aldrich, St. Louis, MO) and incubated with the species-specific secondary antibodies (1:250 or 1:500 dilution). Following secondary antibodies, retinal samples were washed three times, with 4,6-diamidino-2-phenylindole (DAPI) added in the second wash prior to mounting. Retinal sections lacking an obvious foveal depression were stained with Anti-Cone Arrestin (1:250) and 2B2 antibody (1:25) to approximate and assist in localization of the papillomacular bundle. Most confocal images were obtained with 20× oil objective and captured using the Zeiss Axiovert 200 M inverted microscope. Higher magnification images were obtained using a 40× oil objective. Images were processed and acquired using the iVision-Mac™ (BioVision Technologies) software. Brightness and contrast were similarly adjusted in all samples.

### RGC Density and Transmission Electron Microscopy (TEM).

RGC density was quantified for six *OPA1* WT controls (3 females, 3 males; ages 7.0 to 25.5 y) and four *OPA1* heterozygote macaques (3 females, 1 male; ages 7.0 to 28.8 y) on temporal peripheral retinal wholemounts labeled with a rabbit anti-RBPMS antibody (72 h at 4 °C, 1:500; GTX118619, Genetex, Irvine, CA). After incubation with Alexa Fluor 647-conjugated goat anti-rabbit IgG (1:250; 111 605 144, Jackson ImmunoResearch) in 5% NGS and 0.01% Triton X-100 and PBS washes, retinas were mounted on coverslips for imaging. Cell counts from three nonoverlapping images acquired 4 mm from the temporal retinal margin were averaged for each retina using an Axiovert microscope, and representative sequential images were acquired on a Dragonfly spinning disk confocal system.

TEM images were acquired in one *OPA1* WT control (8-y-old male) and one *OPA1* heterozygote macaque (28-y-old female). Optic nerve segments were dissected as ~2 mm cross-sections and fixed in 5 % glutaraldehyde in 0.1 M phosphate buffer, then rinsed in 0.1 M phosphate buffer (three times for 10 min) and the tissues were processed as previously described (52).

### Statistics.

High intereye correlation was observed across all parameters evaluated, thus data from both eyes were averaged and used to represent each macaque. Manual versus automated RNFL measurements were compared in both the *OPA1* heterozygotes and WT controls using a two-way ANOVA with Sidak’s multiple comparison test. A linear regression was used to assess manual RNFL measurements in relation to age and IOP. A Student’s *t* test or Mann–Whitney comparison was applied to analyze differences between WT individuals and their *OPA1* age-matched counterparts for the other outcome measures, as well as to compare optic disc area between age-matched WT males and females. All statistical analyses were carried out using GraphPad Prism version 9.3.1, with significance set at *P* < 0.05.

## Supplementary Material

Appendix 01 (PDF)

## Data Availability

Anonymized data underlying all analyses and supporting the findings presented in this manuscript are available in a single Excel workbook (All_data_Anonymized_25Nov25.xlsx) deposited in the Figshare repository and are publicly accessible at https://doi.org/10.6084/m9.figshare.30716000 (53). The workbook contains the measurements used for all main figures and tables, including optic nerve head imaging and clinical variables, peripapillary retinal nerve fiber layer thickness, pattern electroretinography outcomes, histologic retinal ganglion cell density estimates, *OPA1* genotype information, and fundus and optic nerve assessments. No custom software was created for this study; statistical analyses were performed using GraphPad Prism version 9.3.1 as described in the *Materials and Methods*.
