# Supplementary material for: Rhesus macaques with an OPA1 mutation demonstrate features of autosomal dominant optic atrophy
Source: Proc Natl Acad Sci U S A. 2026 Apr 15;123(16):e2509165123. doi: 10.1073/pnas.2509165123 (PMC13099570; doi:10.1073/pnas.2509165123)
Supplement: Supplementary file 1 — Appendix 01 (PDF) [file pnas.2509165123.sapp.pdf]

Supplementary Information

Supplementary Figures

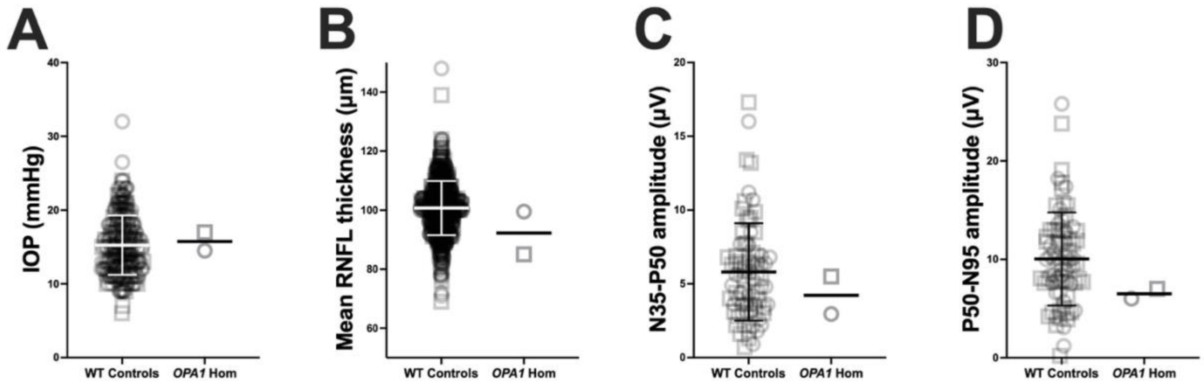

Supplementary Figure 1. *OPA1* homozygous macaques show structural and functional changes consistent with autosomal dominant optic atrophy. Panels show the two homozygotes relative to all wildtype (WT) controls: **A**, intraocular pressure (IOP); **B**, global circumpapillary RNFL thickness; **C**, pattern ERG N35-P50 amplitude; **D**, PERG P50-N95 amplitude. Symbols denote sex: square male, circle female. Horizontal bars indicate group means with whiskers showing ±SD.

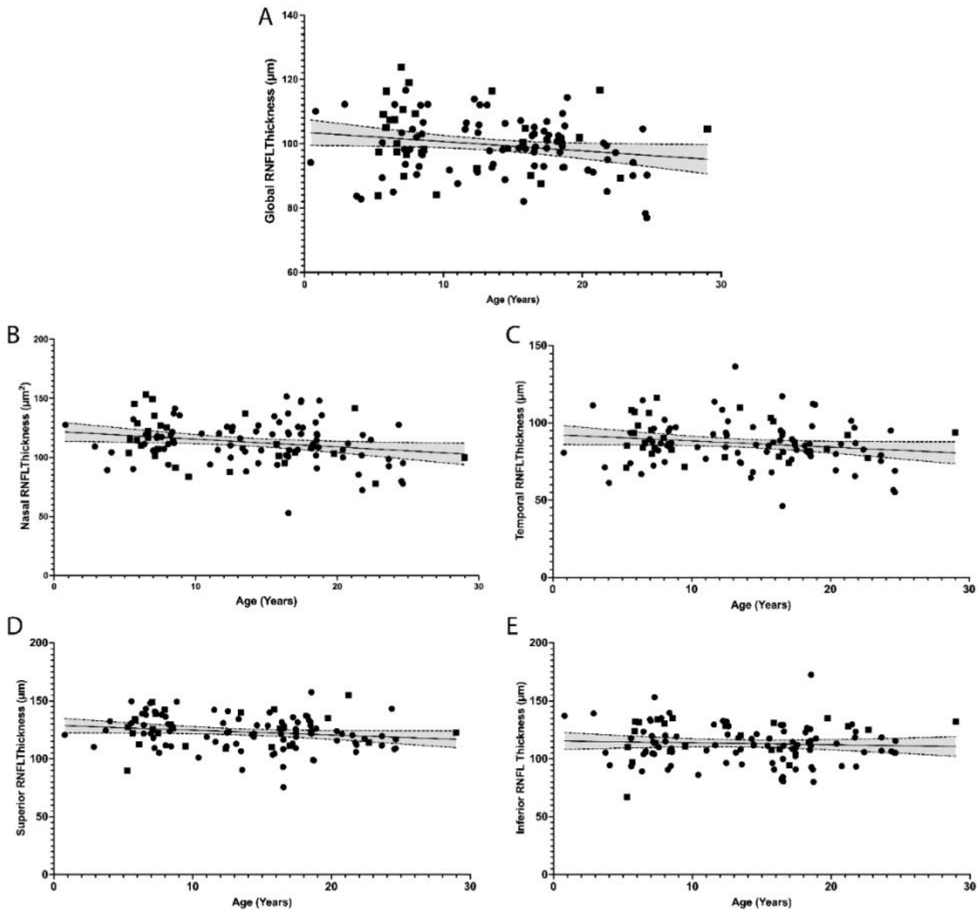

Supplementary Figure 2. Global cpRNFL decreased with age in rhesus macaques, particularly in the nasal quadrant. (**A**) Scatterplot showing relationship between age (years) and RNFL thickness (µm) in 113 rhesus macaques. The RNFL thickness significantly decreased by 0.30 µm per year increase of age (*P*=0.023). (**B-E**) Scatterplots show the relationship between age (years) and the thickness of each quadrant (µm). (**B**) Nasal RNFL significantly decreased by 0.65 µm per year increase of age (*P*=0.019). (**C**) Temporal RNFL decreased by 0.41 µm per year increase of age (*P*=0.067). (**D**) Superior RNFL decreased by 0.41 µm per year increase of age (P=0.066). (**E**) Inferior RNFL increased by a 0.16 µm per year increase of age (*P*=0.516). A linear regression line fitted with a 95% confidence interval is shown in grey.

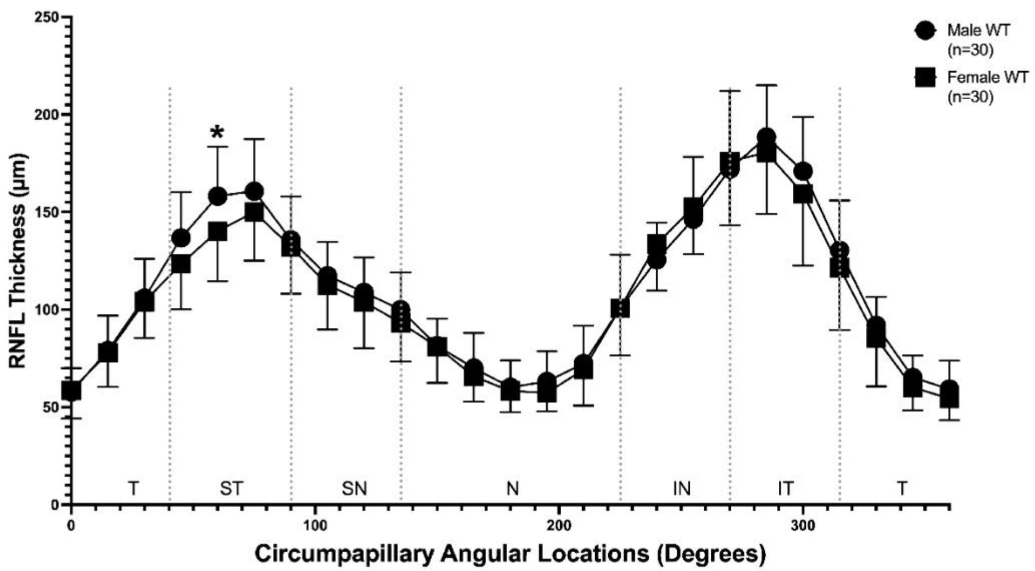

Supplementary Figure 3. In accordance with their representation in the colony, females were overrepresented in the wildtype (WT) study population. To compare differences between sexes, we assessed retinal nerve fiber layer (RNFL) thickness between 30 WT males and 30 age-matched females. Our findings revealed a statistically significant increase in RNFL thickness among females in the superotemporal region (*P*=0.043).

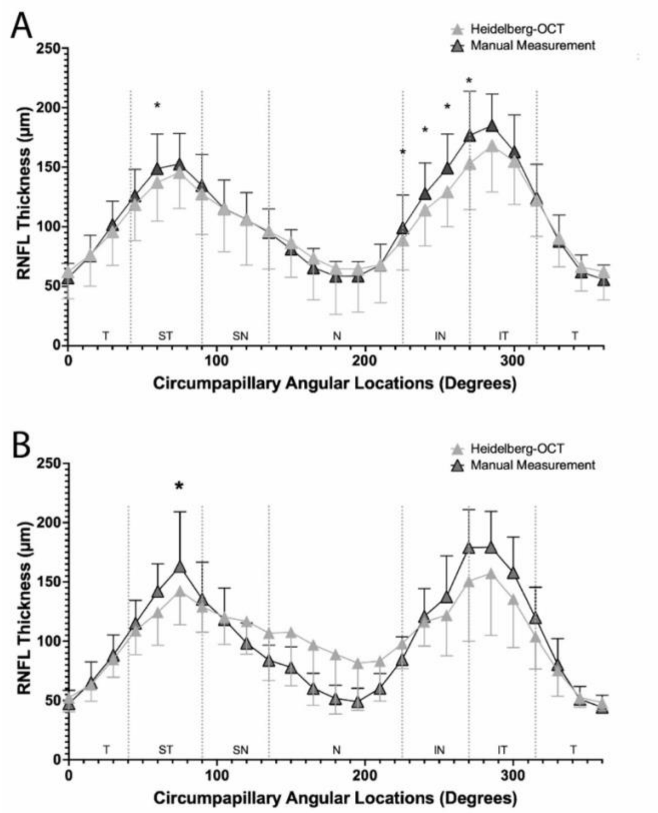

Supplementary Figure 4. Manual measurements significantly differed from automated measurements in both WT controls and *OPA1* heterozygotes. (**A**) A linear regression comparing automated measurements from the Heidelberg Spectralis software and manual measurements of RNFL thickness using ImageJ in 113 WT controls (13.5 ± 6.2 [0.03-29] years of age). Automated and manual measures significantly differed in the 60° superotemporal region and in the inferior regions of 240°, 255°, 270° and 285° (*P<0.05). (**B**) Similarly, *OPA1* heterozygotes also displayed significant differences between the manual and automated measurements, but only in the superotemporal region at 60°. Due to these differences, manual measurements were utilized for the remainder of the study. RNFL thicknesses were compared between measurement technique by two-way ANOVA at all locations using Sidak's multiple comparisons.

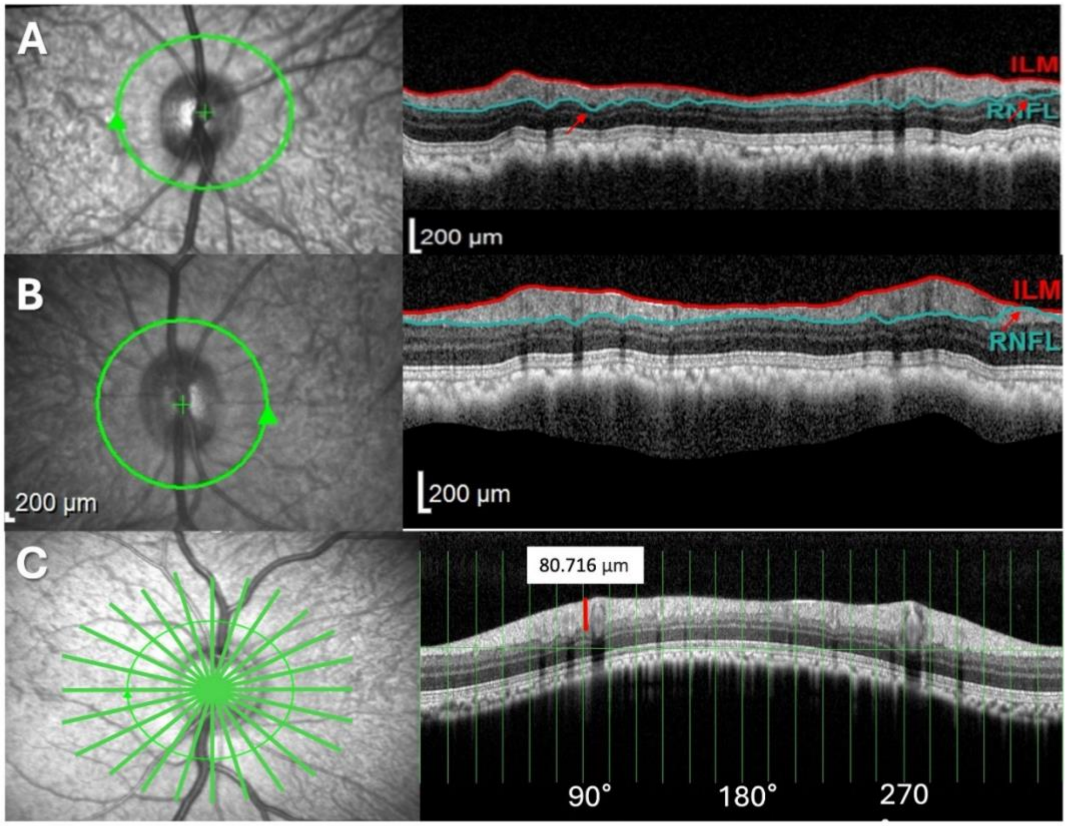

Supplementary Figure 5. The software algorithm of the Heidelberg Spectralis Optical coherence tomography (OCT) was less accurate under diseased conditions. (**A**) An OCT image of the right eye of a 12-year-old *OPA1* heterozygous male shows inaccurate RNFL measurements in the temporal region. There is overmeasurement of the RNFL layer with portions of the GCL included (red arrows) with software categorizing these measurements as within the normal limits of a similarly aged human. (**B**) By contrast, the temporal RNFL measurements in the left eye are undermeasured (red arrow) from the same individual and examination date with the software concluding that the temporal RNFL is outside normal limits of similarly aged humans. In Figure C, the RNFL layer was measured using a 25-point segmentation of a 15-degree circumpapillary area.

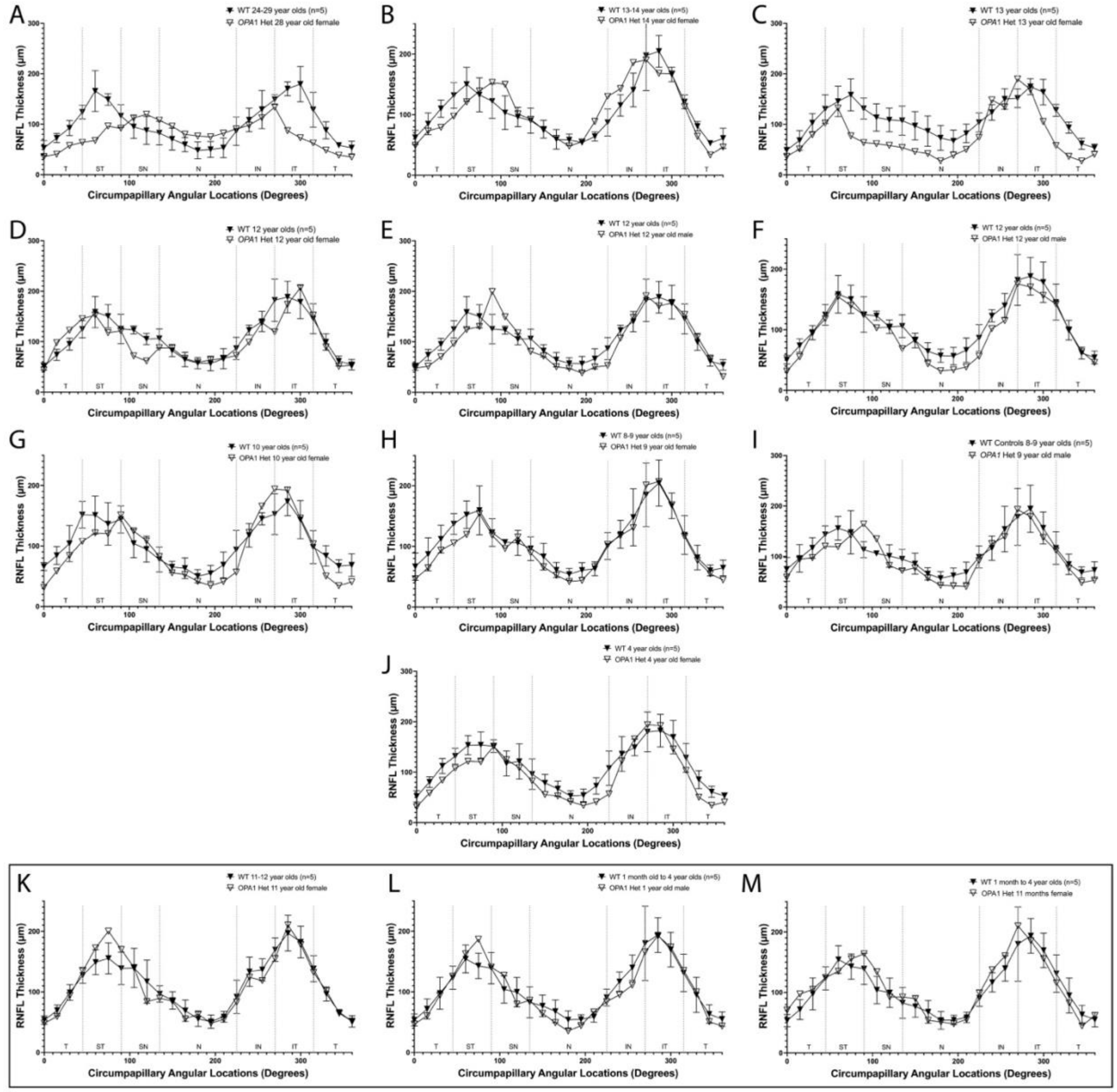

Supplementary Figure 6. The *OPA1* heterozygotes displayed variable changes in RNFL thickness similar to the phenotypic variability observed in humans with ADOA. Ten *OPA1* heterozygotes displayed thinning in at least one region of the peripapillary RNFL in comparison to 5 WT controls (**A-J**), while 3 *OPA1* heterozygotes did not (**K-M**). The 10 *OPA1* heterozygotes with a phenotype were significantly older than the 3 without one at 12.61± 6.21 and 4.35 ± 6.27 years, respectively (P=0.0385). The temporal (n = 7), nasal (n = 7) and superotemporal (n = 5) regions were most commonly reduced while thinning was less commonly observed in the inferotemporal (n = 3), superonasal (n = 3), and inferonasal (n = 2) regions. Manual RNFL measurements were acquired for both the *OPA1* heterozygotes and WT controls.

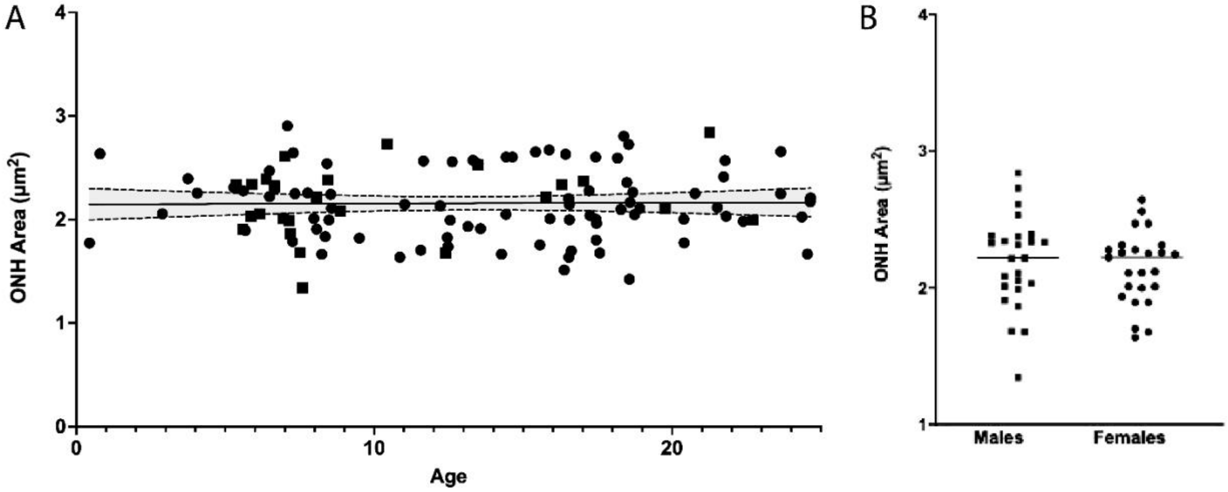

Supplementary Figure 7. Optic nerve head size does not differ by age or sex in normal rhesus macaques. The study included 25 males and 84 females. The age of the females ranged from 0.4 months to 24 years old, with an average optic nerve head (ONH) area of 2.150 (SD = 0.341). The age of the males ranged from 5 to 21 years, with an average ONH area of 2.188 (SD = 0.340). (**A**) The (ONH) area did not significantly differ with age, as determined by a simple linear regression test (*P*=0.8900). (B) The ONH area did not significantly differ between the sexes, and no statistically significant difference was noted (*P*=0.4460).

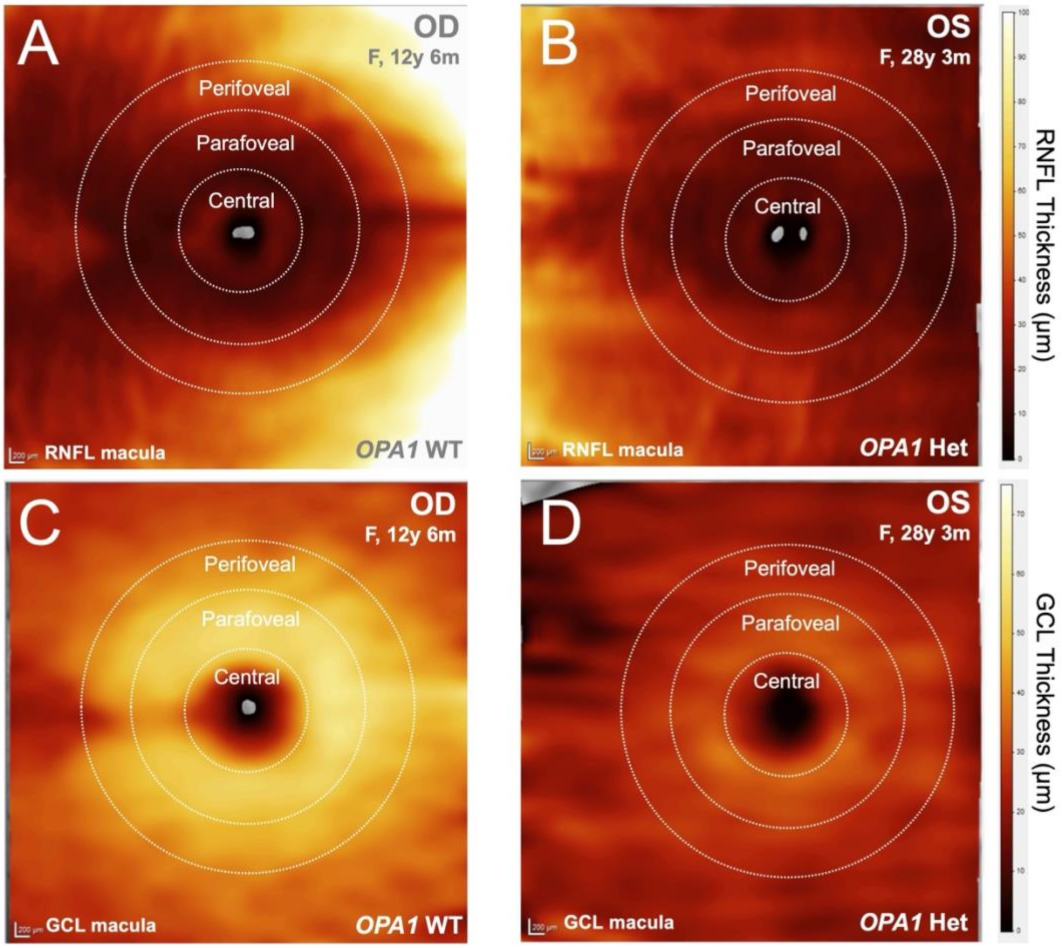

Supplementary Figure 8. Macular retinal layers tend to be thinner in *OPA1* heterozygotes compared with WT controls. A, B: RNFL thickness maps; **C, D:** GCL thickness maps. **A, C**: WT control; **B, D:** *OPA1* heterozygote. Warmer colors indicate greater thickness. **E:** Quantitative ganglion cell complex (GCC = RNFL + GCL + IPL) thickness for the central region. Each point represents one eye (circles = female; squares = male). Horizontal bars show group means with whiskers indicating ±SD. Statistical comparisons between WT and *OPA1* heterozygotes were performed using two-sided Wilcoxon rank-sum tests.

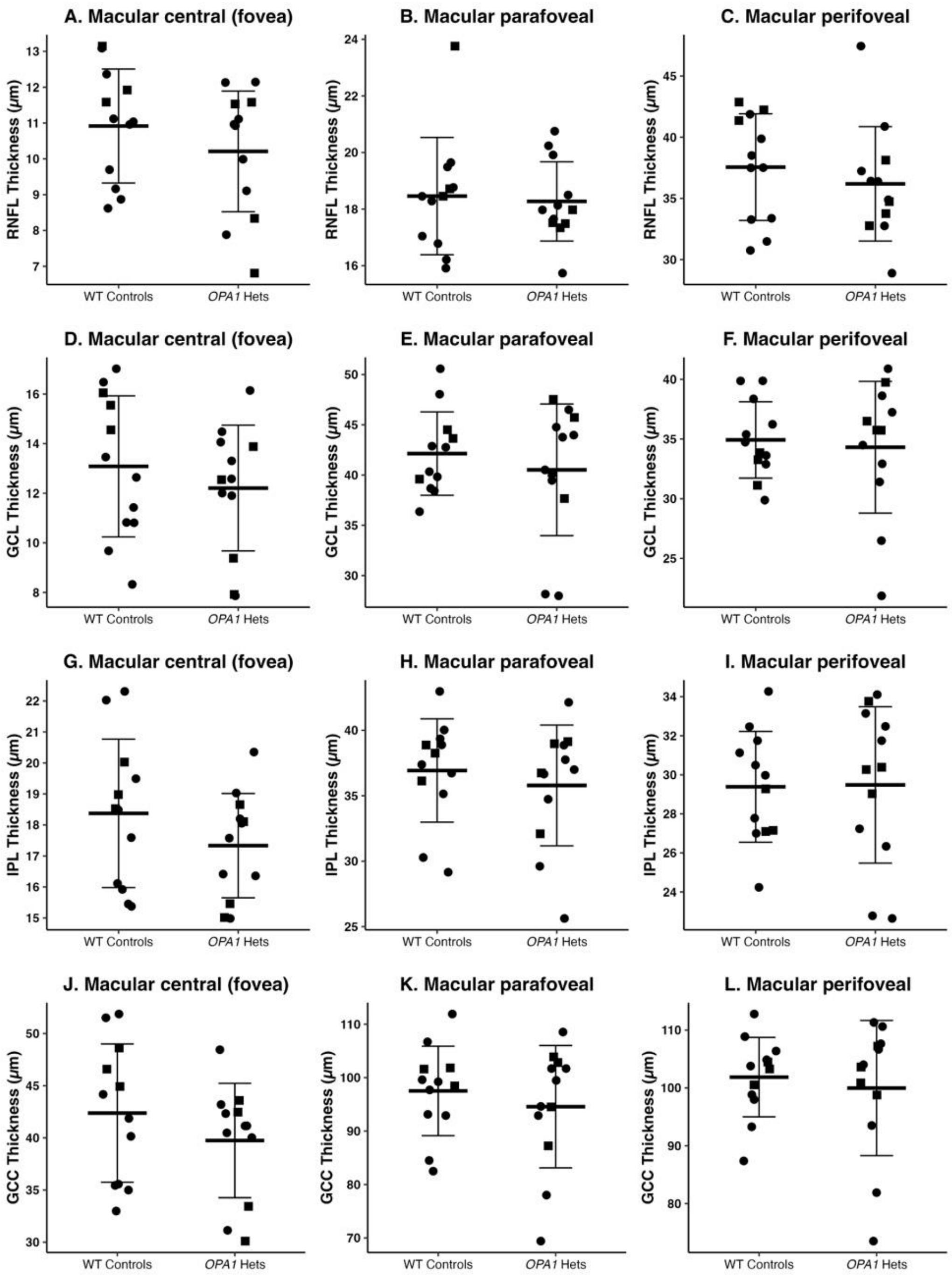

Supplementary Figure 9. Macular retinal layers tend to be thinner in *OPA1* heterozygotes compared with WT controls. A–C, retinal nerve fiber layer (RNFL); **D–F**, ganglion cell layer (GCL); **G–I**, inner plexiform layer (IPL); **J–L**, ganglion cell complex (GCC = RNFL + GCL + IPL). For each eye, central (fovea; **A, D, G, J**), parafoveal (**B, E, H, K**), and perifoveal (**C, F, I, L**) values are shown; parafoveal and perifoveal values are the mean of nasal, temporal, superior, and inferior quadrants. Each point represents one eye (circles, female; squares, male). Horizontal bars indicate group means; whiskers, ±SD. Group comparisons (WT vs *OPA1* Hets) used two-sided Wilcoxon rank-sum tests, but no comparison reached significance.

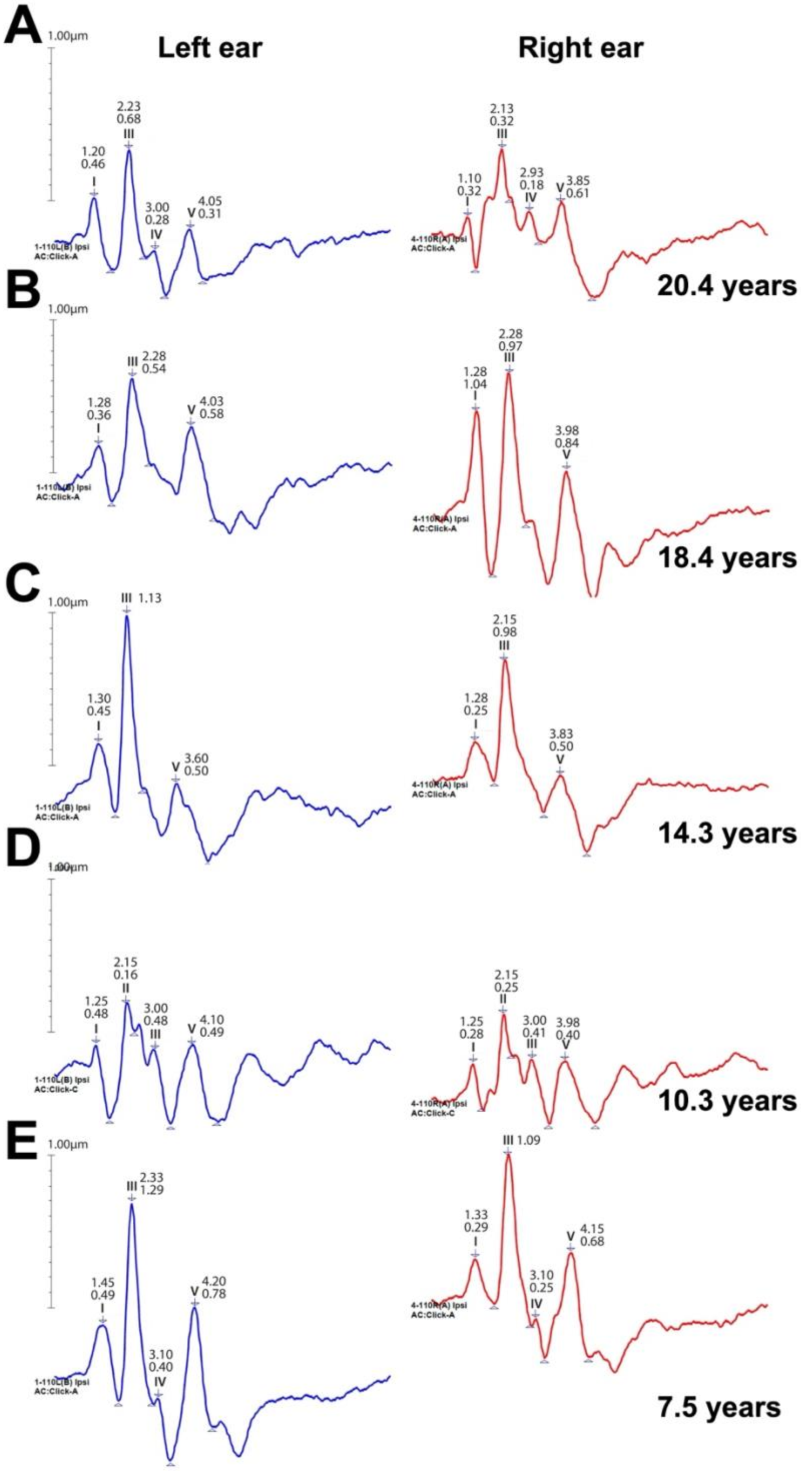

Supplementary Figure 10. Normal brainstem auditory evoked responses (BAER) in *OPA1* heterozygous rhesus macaques. Left-ear (blue) and right-ear (red) responses are shown for five *OPA1* heterozygotes aged 20.4 years (**A**), 18.4 years (**B**), 14.3 years (**C**), 10.3 years (**D**), and 7.5 years (**E**). All macaques showed well-defined waveforms with normal morphology and latencies, consistent with normal auditory function.

Supplementary Data 1

While behavioral visual data is unavailable for the macaques included in the physiological and histological analyses, we had two *OPA1* heterozygotes enrolled on long-term projects including visual tasks. The male was enrolled in the lab projects at the age of 4.1 years until 20.4 years. The female was enrolled in the lab projects from the age of 15.4 years until 21.0 years. Both macaques were extensively trained to participate in behavioral experiments and carried out a variety of tasks that required visual processing across test modalities (e.g., touch screens, using an eye tracker).

Behavioral collected from overt behavioral tasks (Bliss-Moreau and Moadab, 2016; Bliss-Moreau et al., 2021) demonstrated that the male was normal until later in life. Around 18.0 years, his participation in tasks became more variable, until he ceased participating in eye-tracking tasks at approximately 20.0 years. The female performed more variably on tasks from the point of her enrollment and participated inconsistently for her duration of time in the laboratory. She was disenrolled when she would not participate in our standard visual social attention task (Bliss-Moreau, Machado and Amaral, 2013) – generally, the task is well-liked by macaques as indicated in their consistent and prolonged attention to the 30 second videos.

Both subjects did complete a visual attention task in which they were asked to look at monkey faces displaying threat, lipsmack, or neutral faces; method described here: (Santistevan et al., 2024). Thirty rhesus macaques were tested, including both males and females, and ranging in age from 4.1 to 21.9 years (**Supplementary Figure 11**). The *OPA1* heterozygous male was 18.5 years of age and the female was 20 years of age at the time of testing. For the purposes of the present project, total looking time at the faces was summed across all three face categories across all trials. There was no correlation between age and total looking time ($r = -0.24$, $P = 0.20$), nor was there a sex difference in looking time ($F(1,28)=1.73$, $P = 0.20$). Looking times for the male and female *OPA1* heterozygotes were both in the lower third of looking times, with the female in the lowest quartile. Looking times for all subjects are plotted below, organized from longest looking time (a younger female outlier) to the shortest looking time.

The male (but not the female) was tested on our standard social attention task at multiple points across his life (**Supplementary Figure 12**). The task, similar to that detailed here (Bliss-Moreau, Machado and Amaral, 2013) displays 30-second videos of social and nonsocial content that include a variety of content types. Macaques typically engage in this task readily, attending to the videos for nearly the entire 30-second duration and this attention does not decrease with age or repeated presentations of the videos. When tested at 8.3 years of age the male looked for 20.47 s average looking duration at the movies (total sample N=18: M = 24.38, SD = 2.52). When tested at 19.5 years of age, his average looking time dropped to 8.15 seconds. Critically, looking time for the other macaques did not decrease across retest. The sample had shrunk considerably due to natural attrition and so no formal statistics were performed to compare samples (total sample N=8: M = 22.77, SD = 7.15; N=7 with *OPA1* heterozygote dropped: M = 24.85, SD = 4.35).

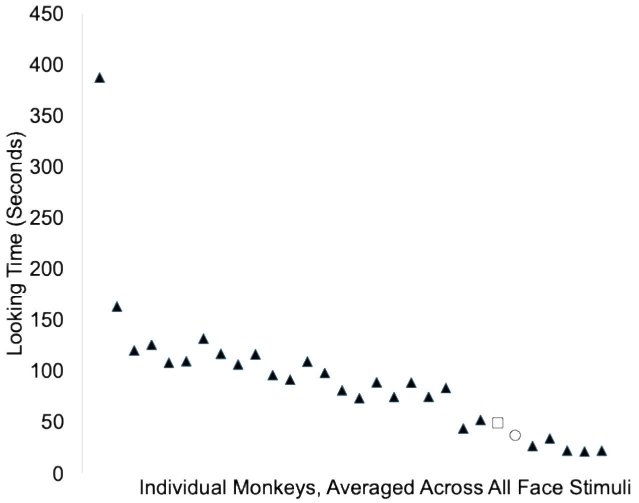

Supplementary Figure 11. Visual attention task results demonstrate shorter looking time in two *OPA1* heterozygous rhesus macaques versus wildtype controls. Total face-looking times are shown for 30 rhesus macaques. Each data point represents the total looking time to faces for each subject, summed across all three face categories and all trials. Subjects are ordered from longest to shortest looking time (left to right); a younger female shows the longest looking time and appears as an outlier at the left of the distribution. The male and female *OPA1* heterozygotes both fall within the lower third of looking times, with the female in the lowest quartile. The *OPA1* heterozygous male is indicated by an open square and the heterozygous female by an open circle.

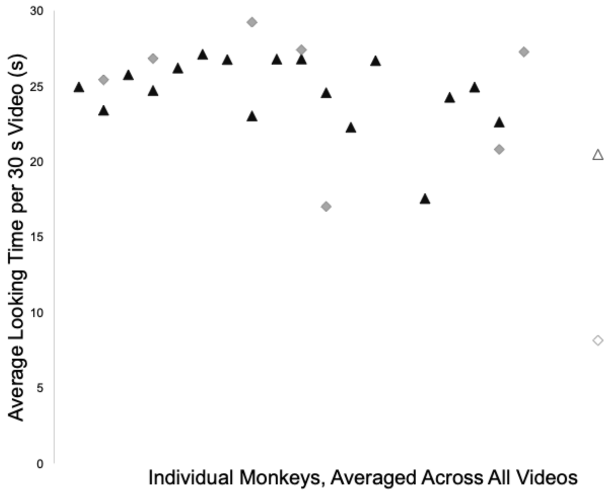

Supplementary Figure 12. Standard social attention task identifies reduced looking time in an *OPA1* heterozygote versus stable looking times in wildtype rhesus macaques. The task was performed at Time 1 and again approximately 11 years later (Time 2). Each point represents the mean looking duration per trial at social movies for one subject. At 8 years and 4 months of age (Time 1), the *OPA1* heterozygous male showed an average looking duration of 20.47 seconds. At 19.5 years of age (Time 2), the average looking duration of this OPA1 heterozygous male decreased to 8.15 seconds, whereas looking durations of the other subjects did not show a systematic decrease across test sessions. Time 1 data are shown as black triangles and Time 2 data as gray diamonds for the wildtype rhesus macaques, with the OPA1 heterozygous male indicated by open symbols at the far right.

References:

1. Bliss-Moreau, E., Machado, C.J. and Amaral, D.G. (2013) "Macaque cardiac physiology is sensitive to the valence of passively viewed sensory stimuli.," Plos One, 8(8), p. e71170. doi:10.1371/journal.pone.0071170.
2. Bliss-Moreau, E. and Moadab, G. (2016) "Variation in Behavioral Reactivity Is Associated with Cooperative Restraint Training Efficiency.," Journal of the American Association for Laboratory Animal Science: JAALAS, 55(1), pp. 41–49.
3. Bliss-Moreau, E. et al. (2021) "Anterior cingulate cortex ablation disrupts affective vigor and vigilance.," The Journal of Neuroscience, 41(38), pp. 8075–8087. doi:10.1523/JNEUROSCI.0673-21.2021.
4. Santistevan, A.C. et al. (2024) "See no evil: Attentional bias toward threat is diminished in aged monkeys.," Emotion, 24(2), pp. 303–315. doi:10.1037/emo0001276.
